# Increased mitochondrial fusion allows the survival of older animals in diverse *C. elegans* longevity pathways

**DOI:** 10.1038/s41467-017-00274-4

**Published:** 2017-08-03

**Authors:** Snehal N. Chaudhari, Edward T. Kipreos

**Affiliations:** Department of Cellular Biology, The University of Georgia, Athens, GA 30602 USA

## Abstract

Mitochondria are dynamic organelles that undergo fusion and fission events. Mitochondrial dynamics are required for mitochondrial viability and for responses to changes in bioenergetic status. Here we describe an insulin-signaling and SCF^LIN-23^-regulated pathway that controls mitochondrial fusion in *Caenorhabditis elegans* by repressing the expression of the mitochondrial proteases SPG-7 and PPGN-1. This pathway is required for mitochondrial fusion in response to physical exertion, and for the associated extension in lifespan. We show that diverse longevity pathways exhibit increased levels of elongated mitochondria. The increased mitochondrial fusion is essential for longevity in the diverse longevity pathways, as inhibiting mitochondrial fusion reduces their lifespans to wild-type levels. Our results suggest that increased mitochondrial fusion is not a major driver of longevity, but rather is essential to allow the survival of older animals beyond their normal lifespan in diverse longevity pathways.

## Abstract (web summary)

Mitochondria can undergo shape changes as a result of fusion and fission events. Here the authors describe how insulin signalling regulates mitochondrial fusion in *C. elegans*, and show that mitochondrial fusion is necessary, but not sufficient, for longevity of worms with mutations that increase lifespan.

## Introduction

Mitochondria play diverse roles in signaling, physiology, and metabolism^1^. Mitochondrial dynamics regulate the morphology, number, and function of mitochondria to allow adaptation to cellular needs^1^. Mitochondrial fission is required for: mitophagy; the mitotic segregation of mitochondria to daughter cells; and the distribution of mitochondria to subcellular locations, such as neuronal axons^2^. Mitochondrial fusion is required for maintaining mitochondrial membrane potential and respiratory capacity, and to protect against apoptosis in mammalian cells^3^.

Cells adjust mitochondrial morphologies to coordinate between the cellular demand for energy and the availability of resources^3^. Elongated morphology is associated with increased efficiency of ATP production and reduced generation of reactive oxygen species (ROS), while fragmented morphology is linked to reduced ATP production and mitochondrial uncoupling.

Mitochondrial fusion and fission events are tightly regulated and require the activity of evolutionarily conserved GTPases^4^. Mitochondrial fission in yeast, invertebrates, and mammals requires the dynamin-related protein DNML1 (Dnm1 in budding yeast and DRP-1 in *C. elegans*). Mitochondrial fusion requires mitofusins for outer membrane fusion (MFN1 and MFN2 in mammals, Fzo1 in budding yeast, and FZO-1 in *C. elegans*), and inner membrane fusion (OPA1 in mammals, Mgm1 in budding yeast, and EAT-3 in *C. elegans*)^4–6^. In mammalian cells, overexpression of the outer membrane fusion proteins MFN1 and MFN2 can lead to either clustering of spherical mitochondria^7–9^ or elongated mitochondria^9, 10^. It is likely that the different outcomes result from differences in the level of expression, as it was shown that modest overexpression of the inner membrane fusion protein OPA1 induces mitochondrial fusion, while higher levels of expression induce fragmentation^11^. In *C. elegans*, the overexpression of either the outer membrane mitofusin FZO-1 or the inner membrane fusion EAT-3 was reported to induce mitochondrial fragmentation^6^; however, in light of the mammalian results, the failure to generate elongated mitochondria could have resulted from excessive expression levels.

Mitochondrial dysfunction, impaired energy homeostasis, and increased production of ROS are associated with aging in both invertebrates and vertebrates^12^. In large-scale *C. elegans* RNA interference (RNAi) screens for genes regulating lifespan, one of the largest classes of genes were those encoding mitochondrial proteins affecting the electron transport chain (ETC)^13^, the RNAi depletion of which can either shorten or extend lifespan. Lifespan extension in response to ETC impairment occurs, at least in part, from the activation of the mitochondrial unfolded protein response (UPR^mt^)^14^. In this study, we present evidence that increased mitochondrial elongation, which occurs independently of UPR^mt^, allows the survival of older animals in diverse longevity pathways.

Insulin/IGF-1 signaling (IIS) is an evolutionarily conserved pathway that controls lifespan^15^. In *C. elegans*, IIS reduces lifespan predominantly by inhibiting the activity of the FOXO transcription factor DAF-16 via inhibitory phosphorylation that blocks its nuclear localization. In the absence of IIS activity, DAF-16 enters the nucleus and regulates gene expression to extend lifespan.

In this study, we describe a pathway for the control of mitochondrial fusion in *C. elegans* that is regulated by IIS and a cullin-RING ubiquitin ligase (CRL). CRLs are multisubunit ubiquitin ligases (E3s) that ubiquitylate substrate proteins to induce proteasome-mediated degradation or post-translational regulation^16, 17^. CRLs include a cullin protein as a scaffold, a RING finger protein, which binds the ubiquitin-conjugating enzyme, a substrate receptor that binds the substrate, and (generally) an adaptor that links the substrate receptor to the core complex. Substrate receptors are variable components, and core CRL complexes function with multiple substrate receptors. The binding of a different substrate receptor to the core CRL complex changes the substrates that are targeted and the cellular function. The most widely studied class of CRLs contains the cullin CUL1 and is designated SCF for its core components: the Skp1 adaptor; CUL1; and F-box protein substrate receptors.

The CAND1 protein functions as an exchange factor for CRL substrate receptors^18–20^. CAND1 affects the steady-state levels of different substrate receptors with the core CRL components^18–20^. In diverse organisms, the loss of CAND1 selectively affects a subset of CRLs, suggesting that certain substrate receptors are particularly reliant on CAND1 activity^16, 17^.

Here, we describe a *C. elegans* mitochondrial fusion pathway that is regulated by CAND-1, the E3 SCF^LIN-23^, and IIS. We show that this pathway is responsible for an increase in elongated mitochondria in response to physical exercise. Additionally, we show that increased levels of elongated mitochondria are associated with diverse lifespan extension pathways, and that the increase in mitochondrial elongation is required to allow the survival of older animals during extended lifespans.

## Results

### The mitochondrial protease *spg-7* is a *cand-1* suppressor

Inactivation of *C. elegans cand-1* results in developmental and morphological defects, including impenetrant embryonic and larval arrest, developmental delays, altered cell divisions, and morphological defects^21^. To identify CAND-1 molecular pathways, we isolated a genetic suppressor mutation, *ek25*, that suppresses multiple *cand-1(tm1683)* loss-of-function phenotypes (Supplementary Table 1). *ek25* was identified as an insertion mutation in an intron of the *spg-7* gene and the 3′ untranslated repeat (UTR) of the *Y47G6A.15* gene (Supplementary Figs. 1 and 2). *Y47G6A.15* is not conserved even in closely related *Caenorhabditis* species. *spg-7* is the ortholog of the mammalian mitochondrial m-AAA protease *AFG3L2* ^22^. RNAi depletion of *spg-7* in *cand-1* mutants enhanced *cand-1* mutant phenotypes, and abrogated suppression in *cand-1*; *spg-7(ek25)* animals (Supplementary Table 1). In contrast, RNAi inactivation of *Y47G6A.15* had no obvious effects (Supplementary Table 1).

**Fig. 1:**
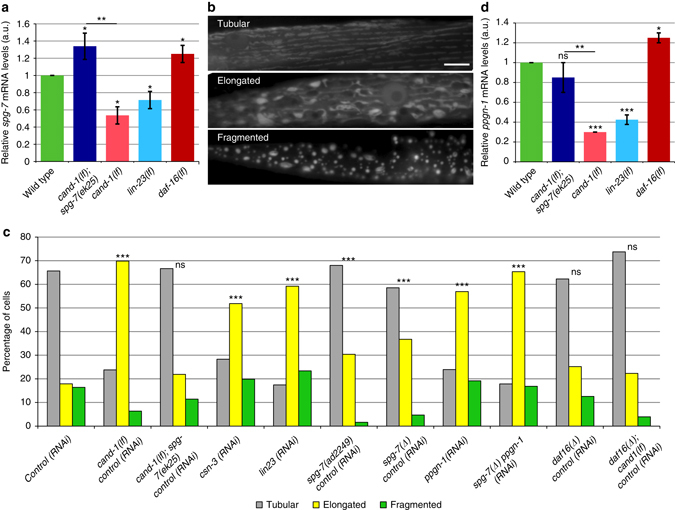
CAND-1/SCF^LIN-23^ regulates mitochondrial shape via *spg-7* and *ppgn-1*. **a** Real-time PCR quantification of *spg-7* mRNA levels in adults of the indicated genotypes, presented in arbitrary units (a.u.) normalized to control *rpl-19* (ribosomal protein L19) mRNA. *Error bars* denote s.e.m. from four biological replicates, each with at least two technical replicates. *P* values were determined by Student’s *t*-test. **b** Images of tubular, elongated, and fragmented mitochondria visualized by mitochondria-targeted GFP expressed in body-wall muscle cells. *Scale bar*, 10 µm. **c** The percentages of muscle cells with tubular, elongated, and fragmented mitochondria for the indicated genotypes and RNAi treatments. All animals were fed either gene-specific RNAi or control RNAi so that the feeding conditions were matched. *P* values were determined by *χ* ^2^-test. Sample size (*n*) of muscle cells from *left* to *right* are: 729; 126; 210; 124, 210; 201; 234; 274; 209; 297; 318; 457. Mitochondrial morphology was scored blinded. **d** Real-time PCR quantification of *ppgn-1* mRNA levels normalized to control *rpl-19* mRNA in adults of the indicated genotypes. *Error bars* denote s.e.m. from three biological replicates, each with at least two technical replicates. *P* values determined by Student’s *t*-test. For all panels*, asterisks* above *bars* denote *P* value comparisons to wild-type/controls; *asterisks* above *lines* denote comparisons under the lines: *P < 0.05; **P < 0.01; ***P < 0.001; ns = not significant

CAND-1 positively regulates *spg-7* mRNA levels. *cand-1* mutants have lower levels of *spg-7* mRNA than wild type, and the *spg-7(ek25)* mutant allele rescues the decrease in *spg-7* expression in *cand-1* mutants (Fig. 1a and Supplementary Fig. 3). These results suggest that *spg-7(ek25)* acts as a gain-of-function mutation that suppresses *cand-1* mutant phenotypes by restoring *spg-7* expression.

### CAND-1 and LIN-23 regulate mitochondrial morphology

Since the *spg-7(ek25)* suppressor regulates the expression of a mitochondrial m-AAA protease, we wanted to determine whether *cand-1* mutants have a mitochondrial phenotype. To characterize mitochondrial morphology, we used a transgenic strain expressing mitochondria-targeted green fluorescent protein (GFP) in muscle cells^23^. Mitochondrial morphology was scored blinded, and the scoring correlates with a quantitative assessment of mitochondrial area (Supplementary Fig. 4). The majority of wild-type hermaphrodite body-wall muscle cells have longitudinally arrayed tubular mitochondria (Fig. 1b, c). Smaller percentages of muscle cells exhibit elongated mitochondria in an interconnected mesh-like network, or fragmented mitochondria (Fig. 1b, c). Inactivation of *cand-1* significantly increases the percentage of cells exhibiting elongated mitochondria; moreover, this phenotype is rescued by the *spg-7(ek25)* suppressor mutation in *cand-1, spg-7(ek25)* animals (Fig. 1c and Supplementary Fig. 5). Inactivation of the COP9/Signalosome component CSN-3, which, like CAND-1, is required for CRL function^16, 17^, also increased the proportion of elongated mitochondria (Fig. 1c and Supplementary Fig. 5). This suggests that the increased mitochondrial elongation phenotype in *cand-1* mutants arises from loss of CRL activity. The E3 SCF^LIN-23^, which contains the substrate receptor LIN-23, is particularly reliant on CAND-1 for activity^21^. We found that inactivation of *lin-23* similarly reduces *spg-7* expression and has increased levels of elongated mitochondria (Fig. 1a, c and Supplementary Fig. 5).

### CAND-1 and SCF^LIN-23^ regulate mitochondria through DAF-16

*spg-7(ad2249)* loss-of-function and *spg-7(tm2312)* deletion (∆) mutants exhibit mitochondrial elongation, but the proportion of mitochondria with elongated morphology is not as large as that observed in *cand-1(RNAi)* or *lin-23(RNAi)* animals (Fig. 1c and Supplementary Fig. 5). Mammalian m-AAA proteases form hexameric complexes in the inner mitochondrial membrane that exist as hetero-oligomeric complexes of AFG3L2 and SPG7, or homo-oligomeric complexes of AFG3L2^24^. We considered whether the partial mitochondrial fusion phenotype of *spg-7*/AFG3L32 mutants could arise from functional redundancy between SPG-7 and PPGN-1, the *C. elegans* ortholog of mammalian SPG7.

We found that inactivation of *ppgn-1* alone increased mitochondrial fusion, while inactivating both *ppgn-1* and *spg-7* further increased the level of elongated mitochondria, implying that both m-AAA proteases negatively regulate mitochondrial elongation (Fig. 1c). CAND-1 and LIN-23 also promote *ppgn-1* expression (Fig. 1d), suggesting that the increase in mitochondrial elongation in *cand-1* and *lin-23* mutants arises from the failure to adequately express both *spg-7* and *ppgn-1*.

To determine how the *spg-7(ek25)* mutation increases *spg-7* mRNA levels, we looked for transcription factor-binding sites that are affected by the *spg-7(ek25)* mutation. The *spg-7(ek25)* mutation disrupts a consensus DAF-16-binding element (DBE)^25^ within a region confirmed for DAF-16 binding by chromatin immunoprecipitation sequencing (ChIP-seq)^26^ (Supplementary Fig. 2). DAF-16 was also shown to bind to the *ppgn-1* regulatory region^26^. A meta-analysis of DAF-16-responsive genes suggests that DAF-16 can repress gene expression through DBE sites (Methods). *daf-16(mu86)* deletion mutants have elevated levels of *spg-7* and *ppgn-1* mRNA, suggesting that DAF-16 is a transcriptional repressor of both *spg-7* and *ppgn-1* (Fig. 1a, d). Unexpectedly, the reduction of *ppgn-1* mRNA levels observed in *cand-1* mutants was also rescued by the *spg-7(ek25)* suppressor allele. The mechanism for this effect is not known but could involve feedback loop(s) or the co-regulation of *spg-7* and *ppgn-1* expression.

**Fig. 2:**
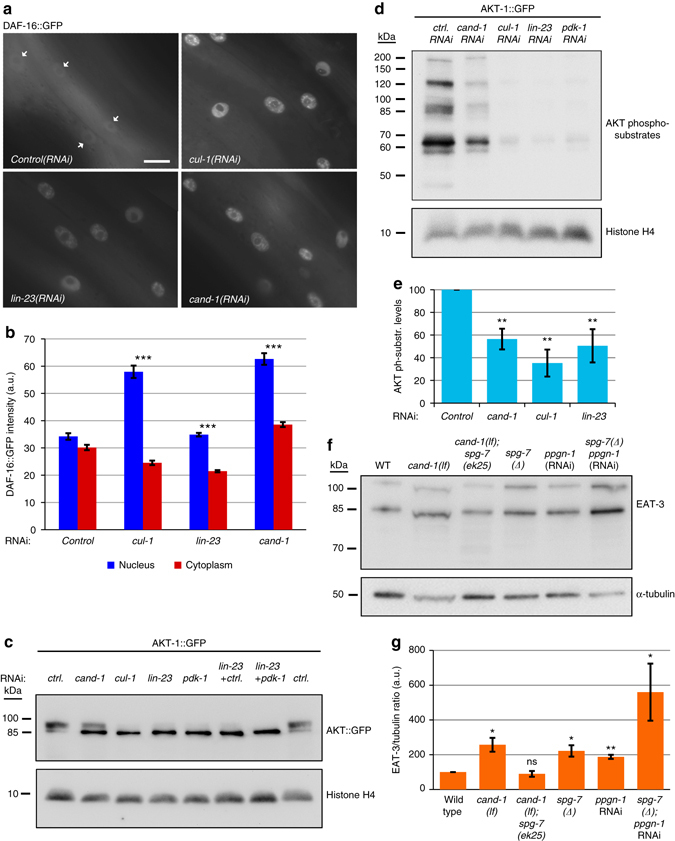
CAND-1/SCF^LIN-23^ activates AKT-1 to inhibit DAF-16 and reduce EAT-3 levels. **a** DAF-16::GFP nuclear localization in body-wall muscle cells for animals with the indicated RNAi treatments. *White arrows* indicate nuclei in the control RNAi image. *Scale bar*, 10 µm. **b** Quantification of the mean level of nuclear and cytoplasmic DAF-16::GFP intensity in body-wall muscle cells. *Error bars* denote s.e.m. *P* values were determined by Student’s *t*-test. Sample size (*n*) of body-wall muscle cells from *left* to *right* are: 44; 61; 102; 87. **c** Western blot with anti-GFP of AKT-1::GFP from whole-animal lysate of L4/young-adult-stage animals treated with the indicated RNAi showing the altered mobility of AKT-1::GFP on SDS-PAGE; anti-histone H4 staining is used as a loading control. Similar results were obtained in two to five biological replicates. **d** Western blot showing staining for an antibody that recognizes AKT phospho-substrates in animals expressing AKT-1::GFP and subjected to the indicated RNAi treatments. Significant decreases in AKT phospho-substrates were observed for *cand-1*, *cul-1*, and *lin-23* RNAi conditions in three to five biological replicates, with *pdk-1* RNAi shown as a control that is known to reduce AKT-1 activity. The identities of the AKT-1 phospho-substrate proteins observed in the western blot are not known. **e** *Graph* showing the levels of AKT phospho-substrate signal relative to control protein bands (anti-tubulin or anti-histone H4) for three to five biological replicates. The control RNAi is set to 100 in arbitrary units. *Error bars* denote s.e.m. *P* values were determined by Student’s *t*-test. **f** Western blot showing EAT-3 protein levels for the indicated genotypes and RNAi treatments. The expected molecular weights of the L-isoform and S-isoform of EAT-3 are 110.1 and 85.5–86.4 kDa, respectively. **g** Quantification of EAT-3 levels normalized to α-tubulin. *Error bars* denote s.e.m. from two to four biological replicates. For all panels, *asterisks* above *bars* denote *P* value comparisons to wild type/controls; *asterisks* above *lines* denote comparisons under the lines: *P < 0.05; **P < 0.01; ***P < 0.001; ns = not significant

We wanted to determine whether CAND-1 and SCF^LIN-23^ promote *spg-7* and *ppgn-1* expression by inhibiting DAF-16 activity. One of the primary mechanisms to control DAF-16 activity is by regulating its nuclear localization^15^. We observed that inactivation of *cand-1*, *cul-1*, and *lin-23* significantly increased DAF-16::GFP nuclear localization, suggesting that CAND-1 and SCF^LIN-23^ normally act to inhibit DAF-16 nuclear localization (Fig. 2a, b). To determine whether the CAND-1-mediated inhibition of mitochondrial fusion is dependent on DAF-16 activity, we combined *daf-16* and *cand-1* loss-of-function mutations. The addition of the *daf-16* mutation rescued the *cand-1*-elongated mitochondria phenotype, suggesting that CAND-1 inhibits mitochondrial elongation by negatively regulating DAF-16 activity (Fig. 1c).

To determine how SCF^LIN-23^ inhibits DAF-16 nuclear localization, we analyzed the kinase AKT-1, which phosphorylates DAF-16 to prevent its nuclear localization^15, 27^. Inactivating *cand-1* or the SCF^LIN-23^ components *cul-1* or *lin-23* leads to the accumulation of a faster-migrating form of AKT-1::GFP without affecting its overall levels (Fig. 2c and Supplementary Fig. 6). AKT-1 is activated by PDK-1 phosphorylation^15, 27^. RNAi depletion of *pdk-1*, or the inhibition of the IIS pathway upstream of PDK-1 and AKT-1, results in the accumulation of a faster-migrating form of AKT-1::GFP that is consistent with the unphosphorylated, inactive form (Fig. 2c)^28^. *pdk-1* RNAi does not produce a further shift in the migration of AKT-1::GFP when combined with *lin-23* RNAi (Fig. 2c). This suggests that the lower molecular weight form of AKT-1::GFP in *lin-23(RNAi)* animals reflects the loss of PDK-1-dependent activating phosphorylation.

To further analyze the activity of AKT-1 in *cand-1(RNAi)*, *cul-1(RNAi)*, *lin-23(RNAi)*, and *pdk-1(RNAi)* animals, we probed whole-animal lysate with a phospho-antibody that detects AKT-phosphorylated substrates. The level of the AKT phospho-epitope was significantly reduced in *cand-1*, *cul-1*, *lin-23*, and *pdk-1* RNAi depletions (Fig. 2d, e). The relatively higher AKT phospho-substrate levels in the *cand-1(RNAi)* animals relative to *cul-1*, *lin-23*, or *pdk-1* RNAi treatments correlated with the reduced effectiveness of *cand-1* RNAi in shifting AKT-1 to the lower molecular weight form in that experiment (Fig. 2c). These results suggest that SCF^LIN-23^ increases AKT-1 activity by promoting its activating phosphorylation.

### SPG-7 and PPGN-1 regulate mitochondrial fusion protein EAT-3

In yeast and mammals, the inner mitochondrial fusion protein, Mgm1 or OPA1, respectively, is cleaved into long (L) and short (S) isoforms, both of which are required for mitochondrial fusion^29, 30^. In mammals, there are two cleavage sites, S1 and S2. S1 is cleaved by the protease OMA1, and S2 is cleaved by the protease YME1L1^29^. *C. elegans* lacks an *OMA1* homolog, and the corresponding S1 cleavage site is not conserved in EAT-3/OPA1. In contrast, *C. elegans* has a *YME1L1* ortholog, *ymel-1*, and its target S2 site is conserved in EAT-3. The expected sizes of EAT-3 are consistent with an L-isoform (after removal of the mitochondrial targeting sequence) and an S-isoform cleaved at the conserved S2 site (Fig. 2f).

**Fig. 3:**
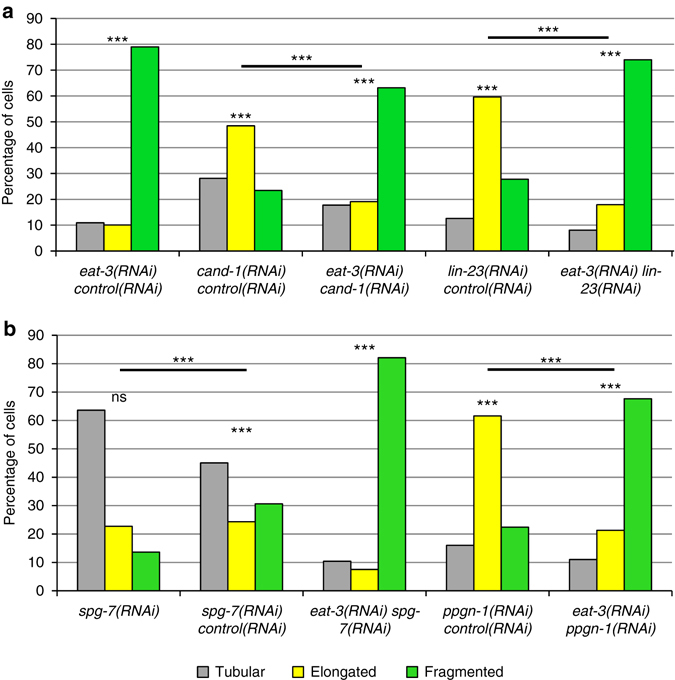
Mitochondrial morphology in *C. elegans* body-wall muscles. **a**, **b** The percentages of muscle cells with predominantly tubular, elongated, or fragmented mitochondria in adult hermaphrodites of the indicated RNAi treatments visualized by mitochondria-targeted GFP expressed in body-wall muscle cells. *P* values were determined by *χ* ^2^-test. Sample size (*n*) of muscle cells from *left* to *right* are: **a** 119, 128, 152, 198, 173; and **b** 151, 111, 173, 125, 136. Mitochondrial morphology was scored blinded. For all panels, *asterisks* above *bars* denote *P* value comparisons to wild type/controls; *asterisks* above *lines* denote comparisons under the *lines*: ***P < 0.001; ns = not significant

Mammalian m-AAA components AFG3L2 and SPG7 can cleave OPA1 when overexpressed or expressed ectopically in yeast^31, 32^. We tested whether inactivating *C. elegans spg-7* and *ppgn-1* affects EAT-3 levels. Co-inactivation of *spg-7* and *ppgn-1* significantly increased the overall level of EAT-3, indicating that both m-AAA proteases negatively regulate EAT-3 levels (Fig. 2f, g). *cand-1* mutants, which have reduced expression of *spg-7* and *ppgn-1*, have higher levels of EAT-3 protein compared to wild type (Fig. 2f, g). Significantly, the *cand-1*; *spg-7(ek25)* strain, which restores expression of *spg-7* and *ppgn-1* mRNA, does not have elevated EAT-3 levels (Fig. 2f, g). The levels of *eat-3* mRNA remain unchanged in the above conditions, indicating that the negative regulation of EAT-3 protein is post transcriptional (Supplementary Fig. 7). It is not known whether the negative regulation of EAT-3 is direct or indirect. EAT-3 appears to function downstream of CAND-1, LIN-23, SPG-7, and PPGN-1, as the *eat-3(RNAi)* mitochondrial fragmentation phenotype is epistatic to the mitochondrial fusion associated with inactivation of these genes (Fig. 3a, b).

The levels of FZO-1::GFP (outer mitochondrial membrane fusion protein) and DRP-1::GFP (inner mitochondrial fission protein) were not affected by inactivation of the mitochondrial fusion pathway genes (Supplementary Fig. 8a, b). We also did not observe a major change in the levels of mitochondria-targeted GFP upon RNAi depletions of the mitochondrial fusion pathway genes (Supplementary Fig. 8c). This suggests that the mitochondrial fusion pathway does not significantly affect the level of mitophagy, as changes in mitophagy affect the level of mitochondria-targeted GFP^33^.

**Fig. 4:**
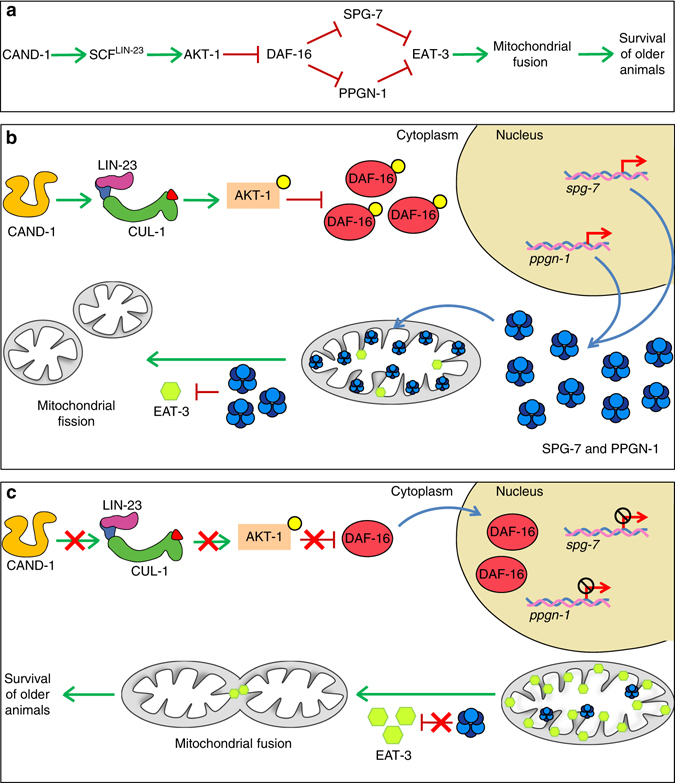
Model for regulation of mitochondrial fusion. **a** Proposed linear pathway for CAND-1 and SCF^LIN-23^ regulation of mitochondrial fusion; see text for description. **b**, **c** *Schematic* of the proposed intracellular pathway regulating mitochondrial fusion and lifespan extension in the presence **b** or absence **c** of CAND-1

Our data support the model shown in Fig. 4, in which CAND-1 activates SCF^LIN-23^, and SCF^LIN-23^ activates AKT-1, thereby reducing DAF-16-mediated transcriptional repression of *spg-7* and *ppgn-1*. SPG-7 and PPGN-1 inhibit mitochondrial fusion, at least in part, by negatively regulating the level of EAT-3.

### Regulation of mitochondrial fusion in physical exertion

**Fig. 5:**
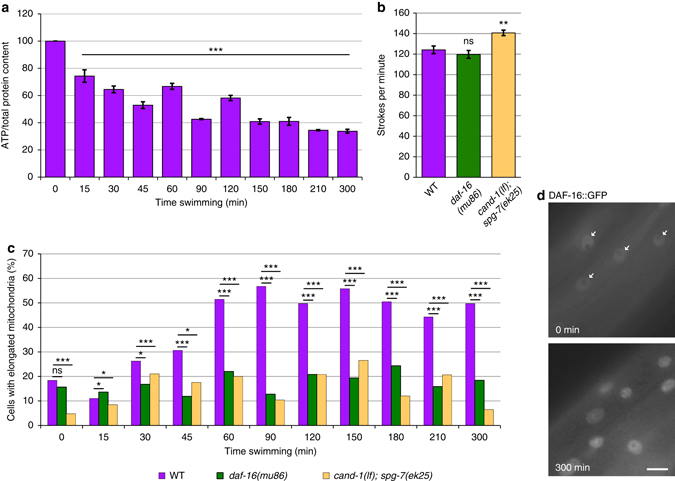
DAF-16 is required for physical exertion-induced mitochondrial fusion. **a** ATP levels decrease during swimming. Graph of ATP levels (normalized to whole-animal protein levels) at the indicated times of continuous swimming. *Error bars* denote s.e.m. from two biological replicates, each with three technical replicates. *P* values were determined by Student’s *t*-test. **b** Average swim strokes per minute of 12 animals each for the indicated genotypes upon induction of swimming behavior. *Error bars* denote s.e.m. *P* values determined by Student’s *t*-test. **c** The percentages of muscle cells with elongated mitochondria in wild type, *daf-16(mu86)*, and *cand-1(tm1683); spg-7(ek25)* animals for the indicated times post induction of swimming behavior. Full distributions of mitochondrial morphology and sample size (*n*) are shown in Supplementary Fig. 9. **d** DAF-16::GFP nuclear localization in body-wall muscle cells for animals at 0 and 300 min post induction of swimming behavior. *White arrows* indicate nuclei in the 0 min image. *Scale bar*, 10 µm. For all panels, *asterisks* above bars denote *P* value comparisons to wild type/controls; *asterisks* above *lines* denote comparisons under the lines: *P < 0.05; **P < 0.01; ***P < 0.001; ns = not significant

Acute physical exertion in mice induces mitochondrial fusion in skeletal muscle cells^34^, presumably to increase the efficiency of ATP production^3^. We wanted to determine whether physical exertion similarly increases mitochondrial fusion in *C. elegans*, and whether the fusion is DAF-16-dependent. To induce physical exertion, we utilized swimming behavior. Animals were kept in a state of constant swimming by gently rocking them in M9 buffer solution that contained OP50 bacteria. Swimming, which manifests as an intense thrashing motion (Supplementary Movies 1 and 2), appears to be vigorous exercise based on the observation that ATP levels decrease during swimming (Fig. 5a). Wild-type animals and *daf-16(mu86)* mutants expressing mitochondria-targeted GFP swam vigorously at an equivalent swimming rate (Fig. 5b and Supplementary Movies 1–3). Wild-type animals showed an increase in elongated mitochondria that reached an elevated plateau at 60 min that persisted through the remainder of the 5-h test period (Fig. 5c and Supplementary Fig. 9a). Significantly, *daf-16* mutants did not exhibit increased levels of elongated mitochondria, implying that DAF-16 is required for this physiological response (Fig. 5c and Supplementary Fig. 9b). Consistently, the nuclear localization of DAF-16::GFP increased in response to swimming in wild-type animals (Fig. 5d and Supplementary Fig. 10). Notably, *cand-1*; *spg-7(ek25)* mutants, which have stabilized *spg-7* and *ppgn-1* expression, exhibited a lack of mitochondrial fusion in response to physical exertion despite a slightly faster swim rate (Fig. 5b, c and Supplementary Fig. 9c). Similarly, we did not observe an increase in mitochondrial fusion in *spg-7(ek25)* mutants, which also swam at a rate equivalent to wild-type animals (Supplementary Fig. 11). This suggests that the DAF-16-mediated inhibition of *spg-7* and *ppgn-1* regulates mitochondrial dynamics in response to physical exertion.

### Mitochondrial fusion is required for IIS lifespan extension

**Fig. 6:**
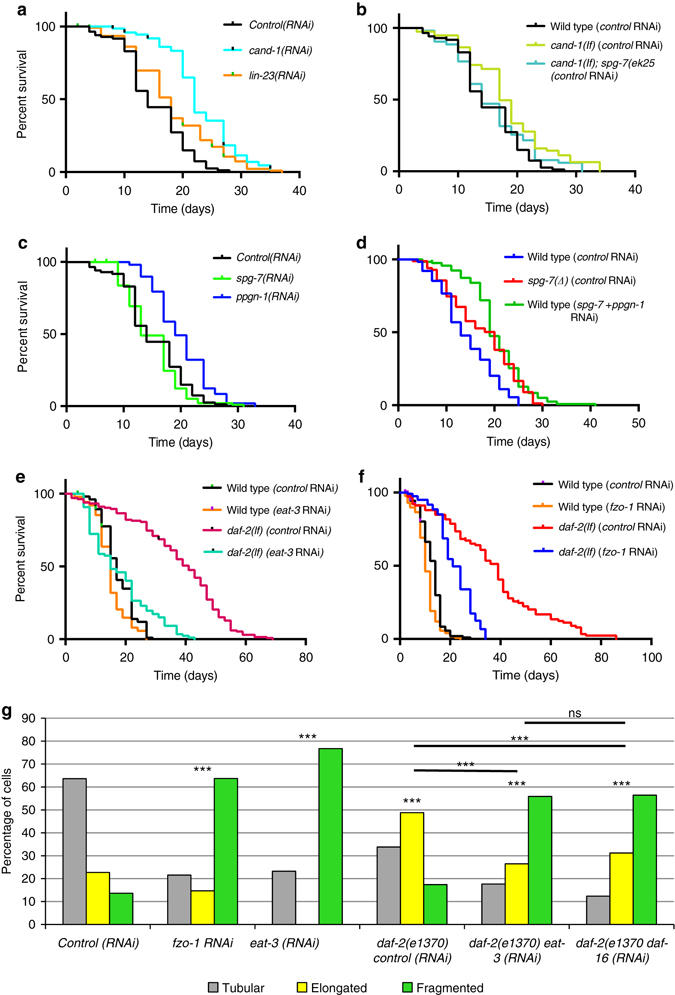
Increased mitochondrial elongation extends lifespans. **a**–**e** *Survival curves* for adults of the indicated RNAi treatments. The wild-type survival curves for **a**–**c** were analyzed at the same time and are shown in each panel for comparison. RNAi depletions of *lin-23* **a**, *cand-1* **a**, *ppgn-1* **c**, and *spg-7* + *ppgn-1* **d** significantly increased mean lifespan. **b** *cand-1* mutants and *spg-7(tm2312)* mutants **d** had increased the mean lifespan relative to wild type, while *cand-1*; *spg-7(ek25)* **c** animals had lifespan comparable to wild type. **e**, **f** *eat-3* RNAi **e** and *fzo-1* RNAi **f** depletions significantly decreased the mean lifespan of *daf-2(e1370)* mutants. All lifespan experiments were performed with four biological replicates. See Supplementary Table 2 for statistics. **g** The percentages of muscle cells with predominantly tubular, elongated, or fragmented mitochondria in adult hermaphrodites of the indicated genotypes/RNAi treatments visualized by mitochondria-targeted GFP expression in body-wall muscle cells. *P* values were determined by *χ* ^2^-test. Sample size (*n*) of muscle cells from *left* to *right* are: 278; 102; 179; 207; 136; 218. Mitochondrial morphology was scored blinded. The wild-type control from Fig. 1c was analyzed at the same time and is shown here for comparison. *Asterisks* above bars denote *P* value comparisons to wild type/control; *asterisks* above lines denote comparisons under the lines: *P < 0.05; **P < 0.01; ***P < 0.001; ns = not significant

DAF-16 is required for the extended lifespan of IIS pathway mutants^27^. Because CAND-1 and SCF^LIN-23^ inhibit DAF-16 nuclear localization, we hypothesized that inactivation of these regulators would extend lifespan. As expected, RNAi depletion of *cand-1* and *lin-23* (begun in the late-L4 larval stage to ensure normal larval development) extended lifespan (Fig. 6a; see Supplementary Table 2 for statistics on lifespan data). Interestingly, the *cand-1*; *spg-7(ek25)* strain, which rescues the *cand-1* increase in elongated mitochondria, had normal lifespan, suggesting that the extended lifespan of *cand-1* mutants requires increased levels of elongated mitochondria (Fig. 6b).

Notably, we observed that increasing mitochondrial fusion independently of DAF-16 produces a modest extension of lifespan. *ppgn-1(RNAi)*, *spg-7(∆)*, and *spg-7* + *ppgn-1* double RNAi animals, all of which exhibit increases in elongated mitochondria (Fig. 1c), had extended lifespan (Fig. 6c, d). In contrast to *spg-7(∆)* mutants, *spg-7(RNAi)* animals do not exhibit increased levels of elongated mitochondria (Fig. 3b), potentially because of the impenetrant effects of RNAi, and consistently had normal lifespan (Fig. 6c).

The mitochondrial fusion pathway includes activating AKT-1 to repress the DAF-16 transcription factor. The IIS pathway has the same downstream pathway, and so mutation of the IIS receptor DAF-2 would also be expected to activate DAF-16-mediated repression of *spg-7* and *ppgn-1* to activate mitochondrial fusion. Consistent with this, *daf-2* mutants exhibit increased levels of elongated mitochondria (Fig. 6g). To determine the extent to which increased levels of elongated mitochondria contribute to the extended *daf-2* mutant lifespan, we inactivated the mitochondrial fusion gene *eat-3* in *daf-2* mutants. Strikingly, *eat-3* RNAi reduced the extended lifespan of *daf-2* mutants to wild-type levels (Fig. 6e). To test an alternate method to reduce elongated mitochondria in *daf-2* mutants, we RNAi-depleted the outer membrane fusion protein FZO-1; moreover, this also significantly reduced the extended lifespan of *daf-2* mutants (Fig. 6f). These results suggest that increased levels of mitochondrial elongation are required for lifespan extension in *daf-2* mutants.

Elongated mitochondria are known to produce ATP more efficiently^3^. We analyzed ATP levels in several key mutants, and found a correlation between increased levels of elongated mitochondria and increased ATP/protein ratios (Supplementary Fig. 12). The *daf-2* mutant is known to have significantly increased ATP levels^35^, and had the highest ATP levels in our assay. Additionally, statistically significant increases in ATP levels were observed in *lin-23(RNAi)*, *cand-1(lf)*, and *ppgn-1(RNAi)* animals, all of which have increased levels of elongated mitochondria. *spg-7(RNAi)* animals, which do not exhibit increased levels of elongated mitochondria (Fig. 3b), did not have significantly increased ATP levels. The observation that *cand-1(lf); spg-7(ek25)* mutants had significantly lower ATP levels than *cand-1(lf)* mutants suggests that a significant portion of the increase in ATP levels in *cand-1(lf)* mutants can be attributed to the reduction of *spg-7* expression. That ATP levels in *cand-1(lf); spg-7(ek25)* remained higher than in wild type may reflect the impact of the loss of CAND-1 on other CRL-regulated processes.

### Physical exertion extends lifespan

**Fig. 7:**
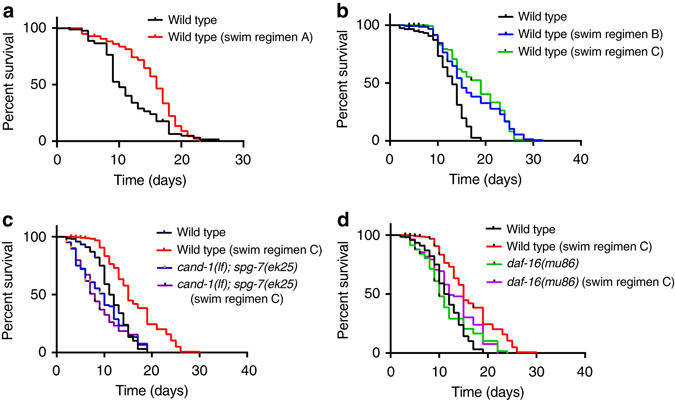
Exercise extends lifespan that is dependent on the mitochondrial fusion pathway. **a**–**d** Survival curves for adults that were kept continuously on agar plates or removed from the plates for brief periods for the described swim regimens. **a** Comparison of wild-type kept on agar plates continuously or subject to swim regimen A (30 min of swimming per day). **b** Test of swim regimens B and C. Swim regimen B was the following swimming times per day on the listed days: 30 min on days 1–5; 25 min on days 6 and 7; 20 min on days 8 and 9; 15 min on day 10; 10 min on day 11; and 5 min on day 12. Swim regimen C was 30 min of swimming on day 1, followed by a reduction in swim time of 2 min per day on subsequent days. **c**, **d** Test of swim regimen C with wild type, *cand-1(tm1683)*; *spg-7(ek25)* **c**, or *daf-16(mu86)* **d**. The wild-type control and wild-type swim regimen C survival curves were analyzed at the same time and are shown in **c**, **d** for comparison. All swimming regimens significantly increased the mean lifespan of wild type **a**–**d**. Swim regimen C did not increase the mean lifespan of *cand-1(tm1683); spg-7(ek25)* **c** or *daf-16(mu86)* mutants **d** relative to the control, non-swimming condition. All lifespan experiments were performed with four biological replicates. See Supplementary Table 3 for statistics

We observed that during swimming, DAF-16 nuclear localization increases, which is associated with lifespan extension. We wanted to determine whether exercise would increase the median lifespan. Making animals swim initially for 30 min per day, followed by consecutive reductions in swim time of 1 min per day, produced significant lifespan extension. However, this swimming regimen (A) caused a rapid drop-off in the viability of older animals (Fig. 7a, see Supplementary Table 3 for statistics). To limit the stress on older animals, we tested two different swimming regimens (B and C) that further reduced the extent of swimming as the animals aged: an initial rate of 30 min swimming/day was tapered via arbitrary reductions (regimen B, Fig. 7 legend), or reductions of two min per day (regimen C). These age-moderated swim regimens produced further lifespan extensions relative to regimen A (Fig. 7b).

Notably, *cand-1; spg-7(ek25)* and *daf-16* mutants, which failed to exhibit increased mitochondrial fusion in response to swimming, did not exhibit lifespan extension in response to swimming (Fig. 7c, d). This suggests that the induction of mitochondrial fusion during exercise contributes to the observed lifespan extension.

Because of how the swim experiments were carried out, the lifespans of the control animals are not directly comparable to the lifespans of control animals in other (non-swim) experiments. Control animals in the swim experiments had shorter lifespans than the control animals in non-swim experiments. Presumably, this difference arose because all animals in the swim experiments (including controls) were kept at room temperature during the swim periods (rather than the lower temperature of 20 °C) and were transferred daily to new plates. Notably, the swim regimens B and C produced a significant extension of lifespan even when compared to control animals from the non-swim lifespan experiments (Supplementary Table 3).

### Mitochondrial fusion is broadly required for longevity

To determine whether mitochondrial fusion is more broadly correlated with longevity, we analyzed the mitochondrial morphologies of animals that exhibit longevity from five distinct mechanisms: *age-1*(*RNAi)* and *pdk-1(RNAi)* animals have extended lifespan from loss of IIS^15^; *eat-1*(*RNAi)* and *eat-6*(*RNAi)* animals have extended lifespan linked to caloric/dietary restriction^36^; *clk-1(RNAi)* extends lifespan because of mitochondrial ETC dysfunction; *glp-1*(*RNAi)* extends lifespan as a result of the loss of the germline^37^; and *vhl-1*, the von Hippel Lindau tumor suppressor ortholog, mutants/RNAi animals have lifespan extension due to deregulation of the hypoxia transcriptional program^38^.

**Fig. 8:**
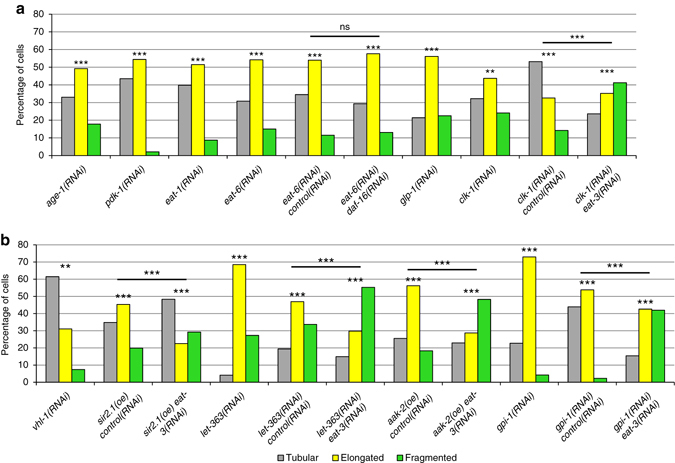
Diverse life extension pathways have increased levels of elongated mitochondria. **a**, **b** The percentages of body-wall muscle cells with tubular, elongated, and fragmented mitochondria in the indicated genotypes, overexpression (oe), and RNAi treatments. *P* values were determined by *χ* ^2^-test. Sample size (*n*) of muscle cells from left to right are: **a** 254, 239, 301, 331, 103, 110, 303, 270, 141, 148; and **b** 148, 181, 120, 121, 98, 114, 153, 122, 260, 264, 143. For all panels, *asterisks* above *bars* denote *P* value comparisons to wild-type/control; *asterisks* above *lines* denote comparisons under the *lines*: *P < 0.05; **P < 0.01; ***P < 0.001; ns = not significant

We analyzed the distribution of mitochondrial morphologies in the seven long-lived strains. Strikingly, six of the seven long-lived mutants/RNAi animals had increased levels of elongated mitochondria, with *vhl-1(RNAi)* animals the lone exception, with predominantly tubular mitochondria (Fig. 8a, b and Supplementary Fig. 5). Caloric restricted mutants, such as *eat-6*, have lifespan extension independent of DAF-16^36^. We found that the increase in elongated mitochondria in *eat-6* mutants was not affected by *daf-16* RNAi, suggesting that mitochondrial morphology in these mutants is regulated through a DAF-16-independent pathway (Fig. 8a). In other organisms, nutrient limitation increases mitochondrial fusion^3^, and it is possible that a similar mechanism operates in *C. elegans*.

**Fig. 9:**
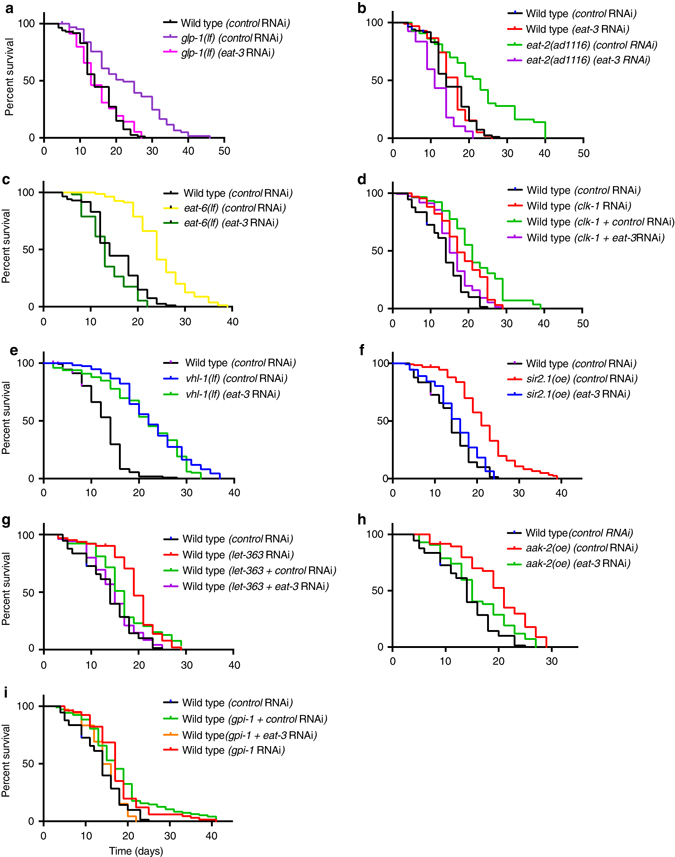
Mitochondrial elongation is required for longevity in diverse mutants. **a**–**i** Survival curves for adults of the indicated genotypes, overexpressions (oe), and RNAi treatments. The wild-type and *eat-3(RNAi)* survival curves are shown in *graphs* for comparison. *eat-3* RNAi significantly reduced the extended lifespans relative of all tested genotypes, overexpression, and RNAi treatments except for *vhl-1* mutants and wild-type control **e**. All lifespan experiments were performed with four biological replicates. See Supplementary Table 2 for statistics

Strikingly, the increase in elongated mitochondria in these diverse mutants is essential for their longevity. RNAi depletion of the mitochondrial fusion gene *eat-3* abolished the extended lifespans of diverse longevity mutants/RNAi: *glp-1(e2141ts)*; *eat-2(ad1116)* and *eat-6(ad467)*; and *clk-1(RNAi)* (Fig. 9a–d). Significantly, *eat-3* RNAi depletion in a wild-type background or in *vhl-1(ok161)* mutants, neither of which have significantly elevated elongated mitochondria, did not affect their lifespans (Fig. 9e). This suggests that *eat-3* RNAi does not have a negative impact on lifespan directly, but rather acts indirectly by reducing the beneficial effects of increased levels of elongated mitochondria.

Elongated mitochondria have decreased production of ROS^3^. We measured mitochondrial ROS levels using the dye MitoSOX Red^39^ in *daf-2*, *glp-1*, and *eat-6* mutants, all of which have elevated levels of elongated mitochondria. Each of these long-lived mutants had reduced ROS levels, and ROS levels increased significantly upon treatment with *eat-3* RNAi to block mitochondrial fusion (Supplementary Fig. 13 and Supplementary Table 4). This suggests that increased levels of elongated mitochondria contribute to reduced ROS levels.

We considered the possibility that the effect on lifespan by the mitochondrial fusion pathway was primarily due to changes in energy levels that were a secondary consequence of the changes in mitochondrial morphology. We analyzed three energy sensors that have an impact on lifespan: LET-363/TOR; SIR-2.1; and AAK-2/AMPK. LET-363/TOR responds to metabolic inputs to regulate lifespan through inhibition of the transcription factors DAF-16, SKN-1, and PHA-4^40, 41^. SIR-2.1 is a NAD^+^-dependent histone deacetylase whose overexpression increases lifespan by activating DAF-16, UPR^mt^, and autophagy^42^. The AMP-activated protein kinase AAK-2 extends lifespan in response to ROS signals and impaired glycolysis, such as inactivation of GPI-1, glucose phosphate isomerase^35, 43^. Overexpression of SIR-2.1 or AAK-2, and RNAi depletion of *let-363*/TOR or *gpi-1*, all exhibited increased mitochondrial elongation (Figs. 8b, c and 9h, i and Supplementary Fig. 5). *eat-3* RNAi reduced the extended lifespan of these animals, suggesting that the elongated mitochondrial morphology contributes to their lifespan extension (Fig. 9f–i and Supplementary Fig. 5).

**Fig. 10:**
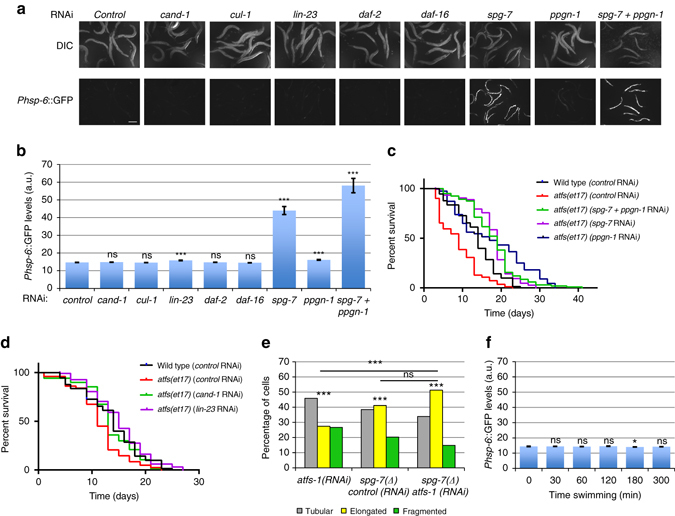
The UPR^mt^ is not involved in the mitochondrial fusion pathway. **a** Representative images of the UPR^mt^ marker *Phsp-6*::GFP in L4/young-adult-stage animals for the indicated RNAi treatments. Scale bar, 200 µm. **b** Quantification of *Phsp-6*::GFP signal for the RNAi treatments shown in **a** for 20 animals. *Error bars* denote s.e.m. *P* values were determined by Student’s *t*-test. **c**, **d** Survival curves for *atfs-1(et17)* gain-of-function mutants with the indicated RNAi treatments; the wild-type survival curve is shown in both *graphs* for comparison. **e** The percentages of body-wall muscle cells with tubular, elongated, and fragmented mitochondria in the indicated genotypes and RNAi treatments. *P* values were determined by *χ* ^2^-test. Sample size (n) of muscle cells from left to right are: 427; 234; 333. **f** Quantification of *Phsp-6*::GFP signal for 20 animals swimming for the indicated times. *Error bars* denote s.e.m. *P* values determined by Student’s *t*-test. The quantification was performed as for **b** and the two *graphs* can be compared directly. No significant differences in *Phsp-6*::GFP were observed relative to 0 min control animals. For all panels, *asterisks* above *bars* denote *P* value comparisons to wild type/controls; *asterisks* above *lines* denote comparisons under the lines: *P < 0.05; **P < 0.01; ***P < 0.001; ns = not significant

The UPR^mt^ links mitochondrial ETC dysfunction during larval stages with lifespan extension in adults^44^. To determine whether the IIS/SCF^LIN-23^-regulated mitochondrial fusion pathway activates the UPR^mt^, we analyzed the induction of the UPR^mt^ reporters *Phsp-6*::GFP^45^. RNAi depletion of *spg-7* is known to induce UPRmt^46^, and, consistently, we observed increased expression of *Phsp-6*::GFP in *spg-7(RNAi)* animals (Fig. 10a, b). Notably, *ppgn-1* RNAi, which induces substantially more mitochondrial fusion than *spg-7* RNAi (Figs. 1c and 3b), did not induce *Phsp-6*::GFP expression (Fig. 10a, b). Additionally, *cand-1*, *lin-23*, and *cul-1* RNAi depletions did not induce *Phsp-6*::GFP, indicating that the mitochondrial fusion pathway does not induce UPR^mt^.

The induction of UPR^mt^ is mediated by the transcription factor ATFS-1, which is required for the expression of *hsp-6* and other UPR^mt^-regulated genes^46^. Counterintuitively, a gain-of-function mutation of ATFS-1, *atfs-1(et17gf)* ^47^, with constitutively activated UPR^mt^, exhibits reduced lifespan, not lifespan extension^48^. We used the *atfs-1(et17)* gain-of-function mutant to ask whether activation of the mitochondrial fusion pathway reduces lifespan further, as would be expected if the pathway further activated the UPR^mt^. We found that RNAi depletion of *cand-1*, *lin-23*, *spg-7*, and *ppgn-1* increased the lifespan of *atfs-1(et17)* mutants, suggesting that the lifespan extension operates independently of the UPR^mt^ (Fig. 10c, d). Induction of UPR^mt^ by loss of *spg-7* is abolished upon the loss of *atfs-1* ^46^. However, we did not observe significant differences in mitochondrial morphology between *spg-7(∆)* mutants and *spg-7(∆)*; *atfs-1(RNAi)* mutants (Fig. 10e). The UPR^mt^ is also not induced upon swimming, unlike the mitochondrial fusion pathway (Fig. 10f). These results suggest that the increase in mitochondrial fusion associated with inactivation of the IIS/SCF^LIN-23^-regulated pathway occurs independently of UPR^mt^.

## Discussion

Our study has revealed a pathway by which the ubiquitin ligase SCF^LIN-23^ and IIS control mitochondrial fusion in *C. elegans* (Fig. 4a). We found that the CRL regulator CAND-1 and SCF^LIN-23^ are required to activate AKT-1. Active AKT-1 inhibits the nuclear localization of DAF-16, which inhibits the expression of the mitochondrial m-AAA proteases SPG-7 and PPGN-1. Decreased expression of SPG-7 and PPGN-1 is associated with increased levels of the mitochondrial fusion protein EAT-3, whose activity is essential for the increased mitochondrial fusion that is observed upon inactivating the pathway components CAND-1, LIN-23, and PPGN-1. Our analysis does not indicate whether SPG-7 and PPGN-1 directly reduce EAT-3 levels or whether the impact on EAT-3 is indirect. Given the additional functions of the yeast and mammalian orthologs of *spg-7* and *ppgn-1*, it is likely that reducing their activity affects multiple mitochondrial pathways^29^. Additionally, SCF E3s are known to target multiple substrates^16, 17^, and we cannot rule out the possibility that CAND-1 and SCF^LIN-23^ have an impact on mitochondria through other pathways.

Our work identifies the SCF^LIN-23^-mediated activation of AKT-1 as a new regulatory mechanism for IIS, for which AKT-1 is a critical component. Currently, it is unclear how SCF^LIN-23^ promotes AKT-1 phosphorylation. Ubiquitylation has been linked to AKT activation in mammalian cells, wherein K63-linked poly-ubiquitylation of AKT is required for its translocation to the plasma membrane, where it is activated^49, 50^. In mammalian cells, the K63 linkage is only detectable with anti-K63 ubiquitin antibodies immediately after stimulation of the relevant signaling pathways, but not in unstimulated cells^49, 50^. We have been unable to detect K63 poly-ubiquitin on immunoprecipitated AKT-1::GFP. However, the inability to rapidly induce signaling in intact animals limits the implications of this negative result.

In mouse skeletal muscle cells, physical exercise increases mitochondrial fusion^34^. We have shown a similar result in *C. elegans* using swimming as a source of exercise. *C. elegans* swimming is associated with a reduction in the level of ATP, indicating that the “thrashing” swim strokes involve a greater expenditure of energy than the normal sinusoidal movement on plates. Swimming induces an increase in the level of elongated mitochondria, and this increase appears to be controlled by the IIS/SCF^LIN-23^-regulated pathway, as the increase in elongated mitochondria is blocked in *daf-16* mutants as well as *cand-1*; *spg-7(ek25)* mutants, which are no longer responsive to DAF-16-mediated inhibition of *spg-7* expression.

In rodents, modest physical exercise correlates with increases in mean lifespan and healthspan^51^, and we have found that exercise regimens increase *C. elegans* lifespan. The lifespan extension in response to exercise is not observed in *daf-16* mutants or *cand-1*; *spg-7(ek25)* mutants, which are unable to activate the IIS/SCF^LIN-23^ pathway to increase mitochondrial fusion, suggesting that increased mitochondrial elongation positively contributes to exercise-induced longevity.

We observed an increase in elongated mitochondria in diverse longevity pathways, including: IIS inactivation; caloric restriction; germline depletion; ETC dysfunction leading to UPR^mt^; TOR inactivation; Sirtuin overexpression; AMPK overexpression; impaired glycolysis; and exercise. RNAi depletions of six genes that cause ETC dysfunction leading to extended lifespan were previously shown to increase mitochondrial fusion^52^. These observations suggest that increased levels of elongated mitochondria are broadly associated with longevity.

Notably, the extended lifespans for the longevity pathway mutants with increased mitochondrial elongation were significantly reduced by *eat-3* RNAi, which reduces mitochondrial fusion. *eat-3* RNAi does not affect the lifespans of wild-type animals or long-lived *vhl-1* mutants, neither of which exhibit increased levels of elongated mitochondria. This suggests that inactivation of the mitochondrial fusion protein EAT-3 does not directly reduce lifespan, but rather indirectly reduces lifespan by countering the beneficial effects of elongated mitochondria. RNAi depletion of the outer membrane mitochondrial fusion protein FZO-1 also reduces *daf-2* mutant-extended lifespan.

*ppgn-1* mutants have a substantial increase in elongated mitochondria, yet have only modestly longer lifespans. It has been reported that blocking fission by inactivating DRP-1 reduces^53^ or has no effect on the lifespan of wild-type animals^54^, but increases the extended lifespans of IIS pathway mutants^54^. These observations suggest that increasing mitochondrial elongation, by itself, does not greatly extend lifespan. Rather, increased levels of elongated mitochondria appear to be a requirement for the survival of older animals in diverse longevity pathways. Thus, while increased levels of elongated mitochondria contribute to older animal survival, the precise level of the increase would not be expected to be predictive of the extent of longevity.

Elongated mitochondria have increased mitochondrial efficiency^3^; in addition, we have observed increased ATP levels in mitochondrial fusion pathway mutants that have increased mitochondrial elongation. This leads to the hypothesis that increased mitochondrial efficiency resulting in sufficient ATP production is required in diverse longevity pathways to allow the survival of long-lived adults.

We observed that *vhl-1* mutants do not have increased levels of elongated mitochondria and their extended lifespan is not reduced by *eat-3* RNAi. Interestingly, a large-scale Cas9 inactivation screen in human cells found that inactivation of VHL allowed a bypass of the complete block on mitochondrial respiration^55^. VHL functions as a substrate receptor for the CRL2^VHL^ ubiquitin ligase, which degrades the hypoxia-inducible factor HIF1Α. During hypoxia, the HIF1Α transcriptional response is associated with a major reorganization of energy pathways to allow energy generation in the absence of oxidative mitochondrial metabolism^55^. Thus, unregulated HIF1Α transcriptional activity can functionally replace the requirement for mitochondrial respiration in human cells^55^.

*C. elegans* VHL-1 similarly targets the degradation of the HIF1Α ortholog HIF-1^56^. HIF-1 is required for the lifespan extension of *vhl-1* mutants^38^. If the role of HIF-1 in providing alternate energy pathways under hypoxic conditions is conserved, then *vhl-1* mutants would have increased energy capacity via HIF-1-mediated induction of energy metabolism. It is possible that increased energy capacity is a requirement for survival beyond the normal lifespan, and that most longevity mutants increase energy capacity via mitochondrial elongation, while *vhl-1* mutants utilize HIF-1-mediated transcriptional activity. It will be interesting to determine whether increased mitochondrial fusion and/or energy metabolism contributes to the survival and healthspan of older animals in other species.

## Methods

### *C. elegans* strains

The following *C. elegans* strains were used, with strain designations in parentheses: wild type Bristol (N2), wild type Hawaiian (CB4856), *cand-1(tm1683)* (ET327), *cand-1(tm1683)* outcrossed 8× into Hawaiian strain CB4856 (ET335), *cand-1(tm1683)*; *spg-7(ek25)* (ET329), *lin-23(e1883)/mIn-1* (ET351), *daf-16(mu86)* (CF1038), *spg-7(ad2249)* (DA2249)^57^, *spg-7(tm2312)* (ET352), *daf-2(e1370)* (CB1370), *muEx248* [*pNL209(Pdaf-16*::DAF-16::GFP) + *Podr-1*::RFP], *Pakt-1*::AKT-1::GFP (SP209)^58^, *glp-1(e2141)* (CF1903), *eat-6(ad467)* (DA467), *zcIs14* [*Pmyo-3::*GFP*(mit)*] (SJ4103)^59^, *cand-1(tm1683)*; *zcIS14* (ET353), *cand-1(tm1683)*; *spg-7(ek25)*; *zcIS14* (ET354), *spg-7(tm2312)*; *zcIS14* (ET355), *daf-2(e1370)*; *zcIS14* (ET356), *daf-16(mu86)*; *zcIS14* (ET357), *cand-1(tm1683)*; *daf-16(mu86)*; *zcIS14* (ET358), *spg-7(ek25)*; *zcIS14* (ET359) *atfs-1(et17)* (QC117); *vhl-1(ok161)* (CB5602); *eat-2(ad1116)* (DA1116), *uthEx299* [*aak-2* (genomic aa1-aa321)::GFP::*unc-54* 3′UTR + *Pmyo-2::*tdTomato] (AGD731), *lin-15(n765ts)*; *bcEx665* [*Phsp*::FZO-1::GFP; *lin-15(+)*] (MD2642), *unc-119(ed3)*; *ekEx37*[pPD96.52/*Pmyo-3*::DRP-1::GFP; *unc-119(+)*] (ET537), *geIs3* [*sir-2.1*(+) + *rol-6(su1006)*] (LG394), and *zcIs13*[*Phsp-6::*GFP] (SJ4100).

### RNA interference

Feeding-RNAi constructs (expressed in HT115 bacteria) were obtained from the Ahringer library^60^. RNAi-feeding bacteria were induced in 1 mM IPTG in liquid 2xYT media plus 100 µg ml^−1^ carbenicillin (Gold Biotechnology) for 5–7 h with the exception of *lin-23*, *let-363*, and *atfs-1* RNAi bacteria, which was induced by plating overnight cultures of the *lin-23* RNAi bacteria on 1 mM isopropyl β-D-1-thiogalactopyranoside (IPTG) plus 100 µg ml^−1^ carbenicillin plates. Double RNAi treatments were performed by combining RNAi bacteria at a 1:1 ratio (unless otherwise indicated) using OD_600_ optical densities to quantify the bacteria prior to seeding plates. Unless otherwise indicated, eggs were placed on the RNAi plates and adults from the next generation were analyzed.

### The isolation of the *ek25 cand-1* suppressor mutation

*cand-1(tm1683)* mutants were synchronized as L1-stage larvae by isolating eggs by hypochlorite treatment^61^ and allowing the eggs to hatch overnight in M9 buffer^61^ supplemented with 5 µg ml^−1^ cholesterol. The synchronized L1-stage larvae were grown on NGM plates with OP50 bacteria until the L4/young-adult stage, when they were mutagenized with 0.5 mM N-nitroso-N-ethylurea (ENU)^62^ for 4 h. The mutagenized animals were cultured on NGM plates with OP50 for 1 day, at which time F1 mutant eggs were isolated from the gravid adults and synchronized as L1 larvae. The F1 mutant animals were cultured on NGM plates with OP50 bacteria until they became gravid adults. F2 mutant eggs were isolated from the F1 gravid adults and synchronized as L1 larvae. In all, 10,000 F2 mutant L1 larvae were grown in a 500 ml liquid culture^61^ supplemented with OP50 bacteria for 11 days, and then collected by centrifugation and regrown in fresh liquid culture for 3 days. Propagation in liquid culture is a hardship for *C. elegans*, and this culturing condition selects for healthier *cand-1* suppressors, as *cand-1(tm1683)* mutants have lower brood sizes, impenetrant embryonic lethality, and impenetrant larval arrest^21^. Animals from the liquid culture were placed on 3xNGM plates. Twelve healthy L4 larvae that did not exhibit *cand-1* mutant phenotypes (e.g., no bobtail phenotype) were cloned onto separate NGM plates with OP50 bacteria. One of these cloned animals (*cand-1(tm1683)*; *ek25*) had 100% healthy progeny without visible *cand-1* mutant phenotypes.

### SNP mapping

The *cand-1(tm1683)*; *ek25* strain was outcrossed 10 times into the Hawaiianized *cand-1(tm1683)* strain ET335. The resulting Hawaiianized *cand-1*; *ek25* strain appears to be largely Hawaiian throughout the genome with the exception of the +7.5 to +11.5 region of chromosome V, which contains the *cand-1* gene and remains N2 Bristol. *cand-1(tm1683)* was crossed with the Hawaiianized *cand-1(tm1683)*; *ek25*, and confirmed F1s were allowed to lay eggs. In all, 273 L1 larvae from the F2 generation were transferred onto separate *csn-3* RNAi plates. *cand-1* mutants subjected to *csn-3* RNAi have an enhanced phenotype of 100% arrested embryos; in contrast, *cand-1*; *ek25* is highly resistant to *csn-3* RNAi (Supplementary Table S1). Using the resistance to *csn-3* RNAi as a measure of *cand-1* suppression, we identified 40 “suppressed” and 40 “non-suppressed” plates from the 273 F2 cross progeny. We performed whole-genome SNP mapping using previously described sets of primers^63^. Pooled DNA from the suppressed and non-suppressed progeny were used to generate PCR amplicons that were digested with the Dra I restriction enzyme (as the SNPs differed in the presence or absence of a Dra I site) in order to distinguish Hawaiian from N2 Bristol genomic DNA. We observed an enrichment of Hawaiian regions in the Chromosome I region −4 to −2 in the “suppressed” population (Supplementary Fig. 1a). We then looked at the distribution of Hawaiian vs. N2 regions in the each of the 40 “suppressed” clones (Supplementary Fig. 1b). Most clones were Hawaiian in the −4 to −2 chromosome I region, but 12 clones were heterozygous in parts of this region. The only SNP that was Hawaiian in all clones was −3.18, which narrowed the location of the suppressor to the −3.96 to −2.07 interval. By sequencing SNPs in this region, we narrowed the region containing the suppressor to −3.48 to −3.18 and found a 26 bp insertion mutation in the major intron of *spg-7* (located at −3.22 on chromosome I). The insertion mutation segregated completely with the rescue of the *cand-1(tm1683)* mutant phenotypes through additional outcrossings, suggesting that it is the *cand-1* suppressor mutation.

### DAF-16 and PQM-1 data meta-analysis

DAF-16 has been found to predominantly function as a transcriptional activator via binding regulatory regions through DBE sites^64^. In contrast, genes that are repressed in a DAF-16-dependent manner are predominantly repressed by the PQM-1 transcription factor via binding to DAE sites^25^. However, an analysis of DAF-16-reponsive genes shows that, of 2196 genes repressed in a DAF-16-dependent manner at *p* < 0.01 significance, 563 (26%) were shown to be bound by DAF-16 but not by PQM-1, and 131 of the genes have position-specific affinity matrices affinity scores of 0 for DAE sites but positive scores for DBE sites^25^. This suggests that DAF-16 can repress gene expression independently of PQM-1. The activating *spg-7(ek25)* mutation disrupts a DBE site, and both *spg-7* and *ppgn-1* regulatory regions have been found to be bound by DAF-16 but not by PQM-1^25^. This suggests that *spg-7* and *ppgn-1* are repressed by DAF-16 in a PQM-1-independent manner.

### Real-time quantitative RT-PCR

Total RNA was isolated from whole-animal lysate using TRIzol reagent (Life Technologies) according to the manufacturer’s instructions. RNA was reverse-transcribed into cDNA using the SuperScript III First-Strand Synthesis for RT-PCR kit from Life Technologies, according to the manufacturer’s instructions. The first-strand cDNA was used for PCR amplification of *spg-7* and *ppgn-1*. *rpl-19*, which encodes the large ribosomal subunit L-19, was used as a normalization control. Reverse transcriptase polymerase chain reaction (RT-PCR) was performed using SYBR Green Supermix (Bio-Rad) and analyzed using the CFX Connect Real-Time PCR Detection System (Bio-Rad). The primers used were: *spg-7*, forward: 5ʹ-CCGTTGTCGTTTGAGACACC-3ʹ, reverse: 5ʹ-CGGCGAAGTGCGTTCATTAC-3ʹ; *ppgn-1*, forward: 5ʹ-ATGCTTCTACACCGCTCCAC-3ʹ, reverse: 5ʹ-GTGGAAATCTGCGAGCACT-3ʹ; *eat-3*, forward: 5ʹ-AGAGCATCGAAACCGGATGG-3ʹ, reverse: 5ʹ-GCGTCAGCATAGCTTCTTCG-3ʹ; and *rpl-19*, forward: 5ʹ-CGCGCAAAGGGAAACAACTT-3ʹ, reverse: 5ʹ-CTTGCGGCTCTCCTTGTTCT-3ʹ. mRNA levels were normalized using *rpl-19* mRNA and the relative fold change was calculated using the ∆∆Ct method. The normalized mRNA levels are reported in arbitrary units with the wild-type level set to 1.0.

### Epifluorescence microscopy

RNAi-treated and mutant animals were maintained at 20 °C, and L4-stage animals to young-adult-stage animals were used for imaging. FZO-1::GFP expressing L4-stage animals were subjected to heat-shock treatment at 33.5 °C for 10 h to ensure stable expression prior to imaging. Animals were mounted on slides with 0.5 mM levamisole (Sigma) to induce muscle paralysis. Animals were visualized with a Zeiss Axioskop microscope, and images were taken with a Hamamatsu ORCA-ER digital camera using Openlab 4.0.2 software (Agilent Technologies). Images were processed and analyzed with Adobe Photoshop CS6 or CC software. Matched images were taken with the same exposure and were processed and analyzed identically. Images from 20 animals were analyzed for AKT::GFP (Supplementary Fig. 6b), FZO-1::GFP (Supplementary Fig. 7a), DRP-1::GFP (Supplementary Fig. 7b), and mitochondrial GFP (Supplementary Fig. 7c) levels.

### Western blot analysis

Mixed-stage animals were lysed in SDS sample buffer and used for western blot analysis. The following mouse primary antibodies were used: anti-GFP (GF28R, Thermo Scientific, cat. no. MA5-15256; 1:2000) and anti-α-tubulin (DM1A, Sigma, cat. no. T9026; 1:4000). The following rabbit primary antibodies were used: anti-EAT-3 (1:1000)^5^; anti-AKT phospho-substrate (Cell Signaling, cat. no. 9611 S; 1:1000); and anti-histone H4 (Upstate Biotechnology, cat. no. 07-108; 1:1000). Anti-rabbit-HRP (ThermoFisher Scientific, cat. no. 32460; 1:5000) and anti-mouse-HRP (ThermoFisher Scientific, cat. no. 32430; 1:10,000) were used as secondary antibodies. Chemiluminescence was performed with the Advanced ECL chemiluminescence system (GE Healthcare). Western Blot images were obtained using the Bio-Rad ChemiDoc MP Imaging System. Western blots were analyzed and quantified with Adobe Photoshop CS6 or CC software using non-saturated images with background-level subtracted. Signals of the bands of interest were normalized with α-tubulin or histone H4, and are reported in arbitrary units. Images of the uncropped western blots for Fig. 2c, d, f are shown in Supplementary Fig. 14.

The expected molecular weight of EAT-3 isoforms was determined as follows. The mitochondrial targeting sequence (MTS), which is cleaved from the precursor protein to form the L-isoform, was identified using the MitoProt program^65^. The predicted MTS in EAT-3 (NP495986) encompasses residues 1–59. The predicted L-isoform therefore encompasses amino acids 60–964 of EAT-3. The mammalian S2 cleavage site has been identified between amino acids 217–223 of human OPA1 (NP_570849)^66^. The human S2 cleavage site corresponds to residues 215–221 in EAT-3, cleavage of which would produce an S-isoform of between 216–964 and 222–964 amino acids.

### Lifespan analysis

Survival assays^67^ were performed at 20 °C. Eggs were isolated from gravid adults by hypochlorite treatment and allowed to hatch on RNAi-feeding bacteria plates, with the exception of *cand-1*, *cul-1*, *lin-23*, *clk-1*, and *let-363* RNAi, where wild-type hermaphrodites were transferred to RNAi-feeding bacteria plates as late-L4-stage larvae to ensure that prior larval development was normal. Animals were transferred to fresh plates every alternate day throughout the lifespan study. For all lifespan studies, animals in the L4 larval stage were picked on day 0. Lifespan analysis of wild-type adult hermaphrodites subjected to a swimming regimen was performed by adding 3 ml of M9 buffer to plates, and the plates were placed on a rocking platform at room temperature to prevent the animals from settling down. The daily swim regimens are described in the main text. After swimming, animals were transferred to fresh agar plates with OP50 bacteria. The non-swim control animals were placed at room temperature for the duration of the swim period, and also transferred to new OP50-seeded NGM plates at the same time as swim animals. Animals that crawled off the plate or had ruptured vulvae were censored. Immobile adults were counted as dead when they failed to respond to prodding. *P* values were calculated by the Log-rank (Mantel–Cox) and Wilcoxon tests using GraphPad Prism software (version 6.0).

### ATP quantification

ATP levels were measured using the EnzyLight^TM^ ADP/ATP Ratio Assay Kit (ELDT-100) from BioAssay Systems as per the manufacturer’s protocol with the following modifications. Eggs were isolated from gravid adults by hypochlorite treatment and allowed to hatch on RNAi-feeding bacteria plates, or OP50 plates for mutant animals, and maintained at 20 °C. L4-stage animals were collected by centrifugation and washed three times in M9 buffer +5 mg ml^−1^ cholesterol, flash-frozen in liquid nitrogen, and stored at −80 °C. Frozen samples were boiled for 15 min to release ATP, and centrifuged at 15,000 × *g* to pellet debris. The supernatant was diluted twofold in RNase/DNase-free water, and used for ATP measurement. Protein concentration was measured using the Bradford reagent (Sigma). ATP levels were normalized to protein concentration.

### Analysis of mitochondrial morphology

Adult hermaphrodites expressing mitochondrial matrix-targeted GFP [*Pmyo-3*::GFP(mit)] in their body-wall muscle cells^59^ were imaged for mitochondrial morphology. Cells were categorized as harboring tubular, elongated, or fragmented mitochondria, as described^5^. Mitochondrial morphology was assessed with at least 100 muscle cells analyzed for each condition, and was scored blinded where noted. To quantitatively categorize cells harboring tubular, elongated, or fragmented mitochondria, the two-dimensional areas of mitochondria were measured as previously described^68^. The areas (in pixels) of the five largest mitochondria per cell in ~40 body-wall muscle cells were measured from micrographs using Photoshop software (Adobe).

### Analysis of mitochondrial ROS

Eggs were placed on RNAi plates and L4-stage larvae were used for the analysis of mitochondrial ROS with the dye MitoSOX Red (Life Technologies), which was performed as previously described^39^. Animals were incubated in 10 µM MitoSOX Red in M9 buffer with cholesterol and RNAi bacteria in the dark at 20 °C for 24 h. Animals were washed two times in M9 buffer and incubated in M9 buffer with OP50 bacteria for an hour to clear their intestines of residual dye. ROS levels were analyzed in the posterior pharyngeal bulb by measuring the MitoSOX Red epifluorescence intensity with a 300 ms exposure. Epifluorescence was analyzed with optical filters of 470/40 excitation wavelength and 500 long-pass emission wavelength. Mitochondrial ROS levels were measured in adult hermaphrodites 1 day-post-L4 stage.

### Physical exertion swimming assay

L4-stage larvae and young adults expressing the *Pmyo-3*::GFP(mit) transgene were placed on one 6-cm OP50-seeded NGM plate per time point. To induce the swimming behavior, 3 ml of M9 buffer was added to each plate, and the plates were placed on a rocking platform to prevent the animals from settling down. At each time point, animals were collected by centrifugation, and visualized by epifluorescence microscopy to determine mitochondrial morphology (obtaining data from at least 100 cells). To determine the swim rate, 12 animals were analyzed for the number of swim strokes per minute for each genotype.

### Data availability

The authors declare that all data supporting the findings of this study are available within this article, its Supplementary Information files, or are available from the corresponding author upon reasonable request.

## Electronic supplementary material

Supplementary Movie 1

Supplementary Movie 2

Supplementary Movie 3

Supplementary Information
