# Supplementary material for: Increased mitochondrial fusion allows the survival of older animals in diverse C. elegans longevity pathways
Source: Nat Commun. 2017 Aug 3;8:182. doi: 10.1038/s41467-017-00274-4 (PMC5541002; doi:10.1038/s41467-017-00274-4)
Supplement: Supplementary file 4 — Supplementary Information [file 41467_2017_274_MOESM4_ESM.pdf]

File Name: Supplementary Movie 1
Description: Wild-type *C. elegans* adult hermaphrodites moving on a solid agar surface.

File Name: Supplementary Movie 2
Description: Wild-type *C. elegans* adult hermaphrodites swimming in an M9 buffer solution.

File Name: Supplementary Movie 3
Description: *daf-16(mu86)* mutant *C. elegans* adult hermaphrodites swimming in an M9 buffer solution.

File Name: Supplementary Information
Description: Supplementary Figures, Supplementary Tables, Supplementary References.

a

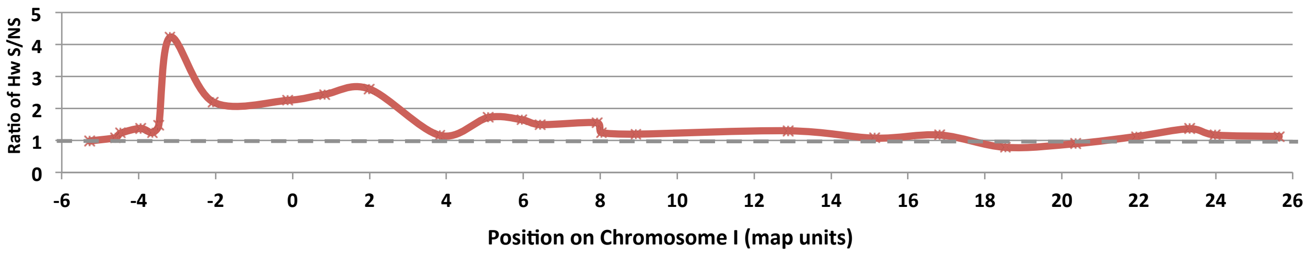

b

Suppressed clones	Characterization of SNPs (listed by genetic map position on Chromosome I)							
	-5.27	-4.61	-4.47	-3.96	-3.18	-2.07	-0.13	+0.83
7	het	Hw	Hw	Hw	Hw	Hw	het	het
9	het	Hw	Hw	Hw	Hw	het	het	het
15	het	Hw	Hw	Hw	Hw	het	het	het
18	het	het	Hw	Hw	Hw	Hw	Hw	Hw
20	het	Hw	Hw	Hw	Hw	Hw	Hw	het
21	het	Hw	Hw	Hw	Hw	Hw	Hw	het
22	het	het	Hw	Hw	Hw	Hw	Hw	het
25	het	het	Hw	Hw	Hw	Hw	Hw	Hw
33	Hw	Hw	Hw	Hw	Hw	het	het	het
35	het	het	het	het	Hw	Hw	Hw	Hw
37	het	het	Hw	Hw	Hw	het	het	het
38	het	het	het	Hw	Hw	het	het	het

Supplementary Figure 1. Identification of the suppressor region in *cand-1*; *ek25*.
(**a**) Ratio of Hawaiian to Bristol (N2) SNPs in the 'suppressed' (S) vs. 'not-suppressed' (NS) progeny for chromosome I from a cross of Hawaiianized *cand-1*; *ek25* mutant and *cand-1* mutant (N2 background). Note that the Hawaiianized *cand-1*; *ek25* mutation was previously outcrossed 10 times to a Hawaiianized *cand-1* mutant strain. This effectively replaced N2 SNPs in the original *cand-1*; *ek25* mutant strain with Hawaiian SNPs. There is a peak of suppression between SNPs at -3.96 and -2.07, suggesting that the *ek25* suppressor mutation is in this region. This SNP mapping was repeated two times. (**b**) Clonal analysis of individual 'suppressed' cross progeny for region -5.27 to +0.83 on chromosome I. SNPs for each progeny clone are marked as heterozygous for Hawaiian and N2 SNPs (het, yellow) or homozygous for Hawaiian SNPs (Hw, green).

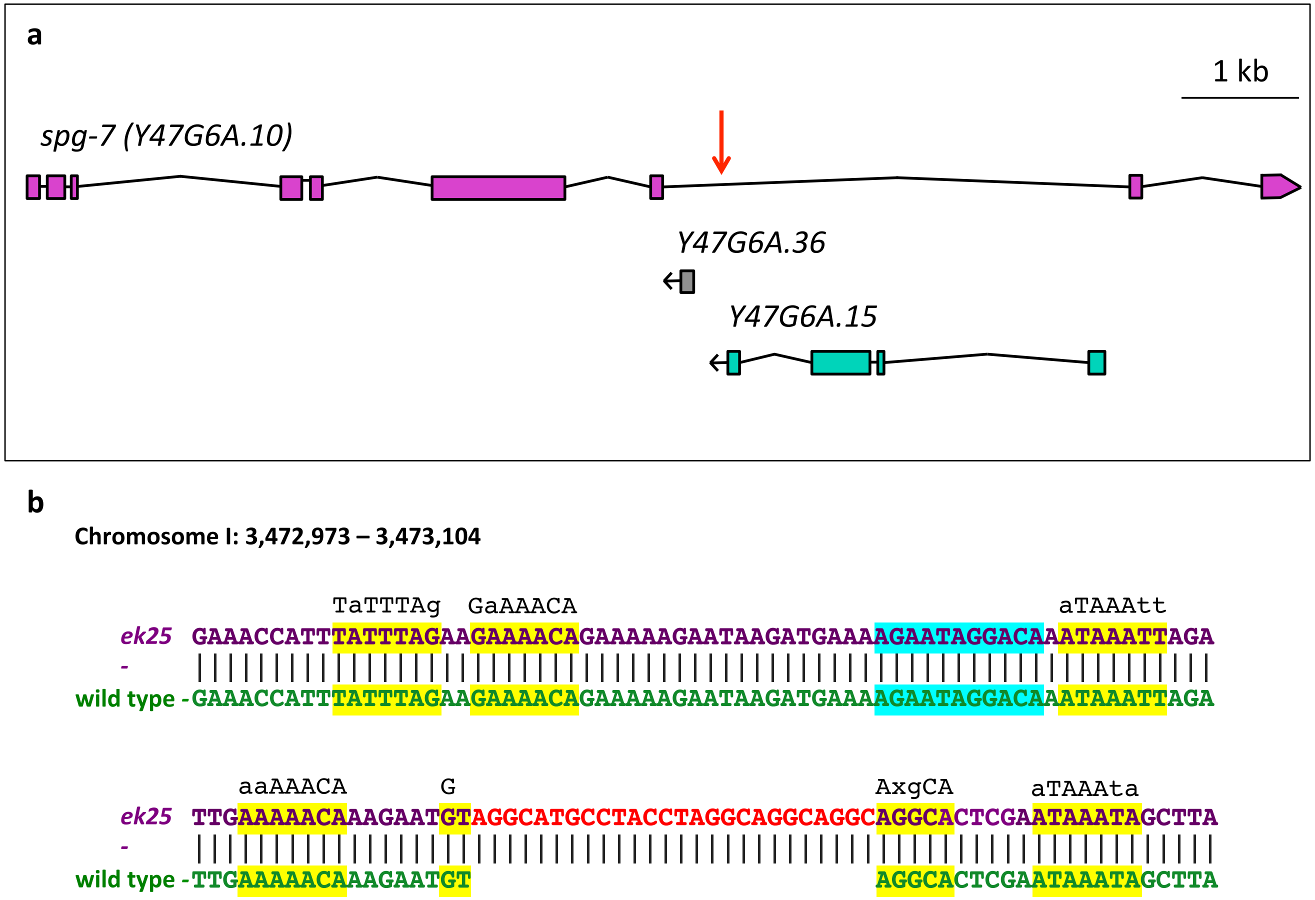

Supplementary Figure 2. The location of the *cand-1*; *ek25* suppressor mutation.
(**a**) Schematic of the region of chromosome I containing the genes *spg-7* (encoding an m-AAA protease), *Y47G6A.15* (encoding a non-conserved protein), and the non-conserved, non-coding RNA *Y47G6A.36*. The location of the *ek25* mutation is marked by a red arrow. (**b**) The *ek-25* allele is a 26 bp insertion (shown in red lettering) that disrupts a predicted DAF-16-binding element (DBE) (highlighted in yellow). This region contains six sequences that differ from the DBE consensus (GTAAACA and TGTTTAC) by one or more nucleotides, with the alternate nucleotide generally the second most abundant nucleotide for the consensus position[1,2]. The DBE matches are highlighted in yellow and listed above the sequence with the dominant nucleotide for a consensus position in upper case, the second most abundant nucleotide in lower case, and nucleotides that do not match the consensus marked by an 'x'. The sequence highlighted in blue was identified as a DAF-16::GFP-bound sequence by ChIP[3].

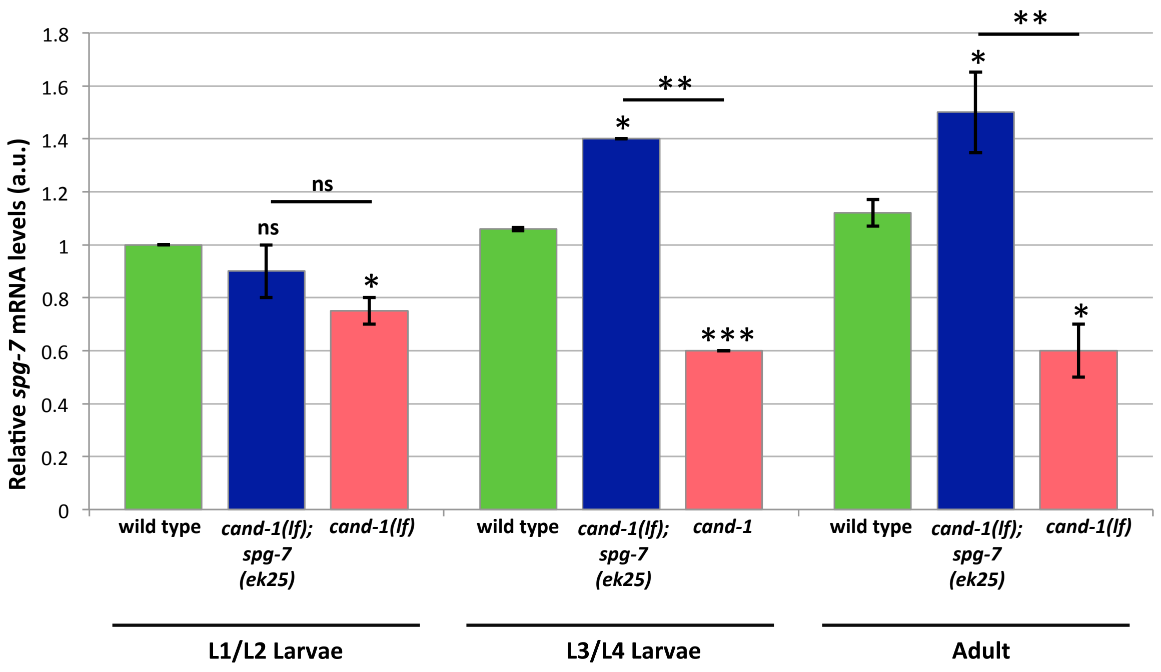

Supplementary Figure 3. Real-time quantitative PCR of *spg-7* mRNA levels.
RT-qPCR quantification of *spg-7* mRNA levels normalized to *rpl-19* control mRNA for L1/L2-stage larvae, L3/L4-stage larvae, and adults of the indicated genotypes. The mRNA levels are reported in arbitrary units with the wild-type level for L1/L2 larvae set to 1.0. Error bars denote standard error of the mean (s.e.m.) from two biological replicates, each with at least two technical replicates. *P* values were determined by Student's t-test. Asterisks above bars denote *P* values for comparisons to wild type; asterisks above lines denote comparisons under the lines: *P<0.05; **P<0.01; ***P<0.001; ns = not significant.

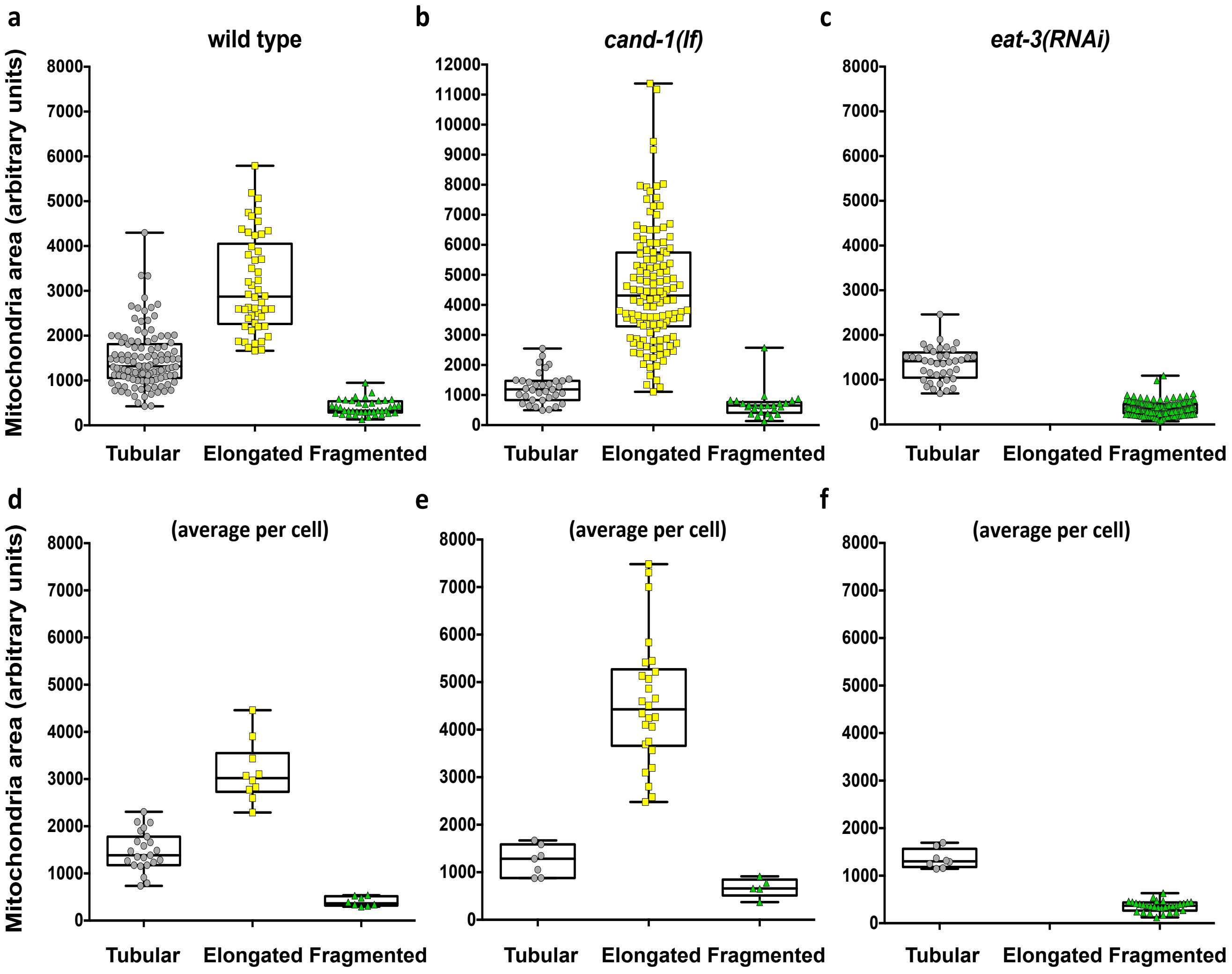

Supplementary Figure 4. Quantification of mitochondrial morphologies.
(**a**-**c**) Scatter plot of the areas of the five largest mitochondria per cell in micrographs of ~40 body wall muscle cells per genotype visualized by mitochondria-targeted GFP expression for wild type (**a**), *cand-1(lf)* (**b**), and *eat-3(RNAi)* (**c**). Note that the categorization of cell mitochondria as tubular, elongated, or fragmented correlates with the areas of the mitochondria in these cells. (**d**-**f**) Scatter plot of the averages per body wall muscle cell of the areas of the five largest mitochondria for wild type (**d**), *cand-1(lf)* (**e**), and *eat-3(RNAi)* (**f**). Boxes represent the s.e.m. range with the mean denoted by a central horizontal line; vertical lines extending above and below the box denote the range. Within each genotype, the tubular, elongated, and fragmented distributions are statistically different; $P < 0.0001$ using Student's t-test.

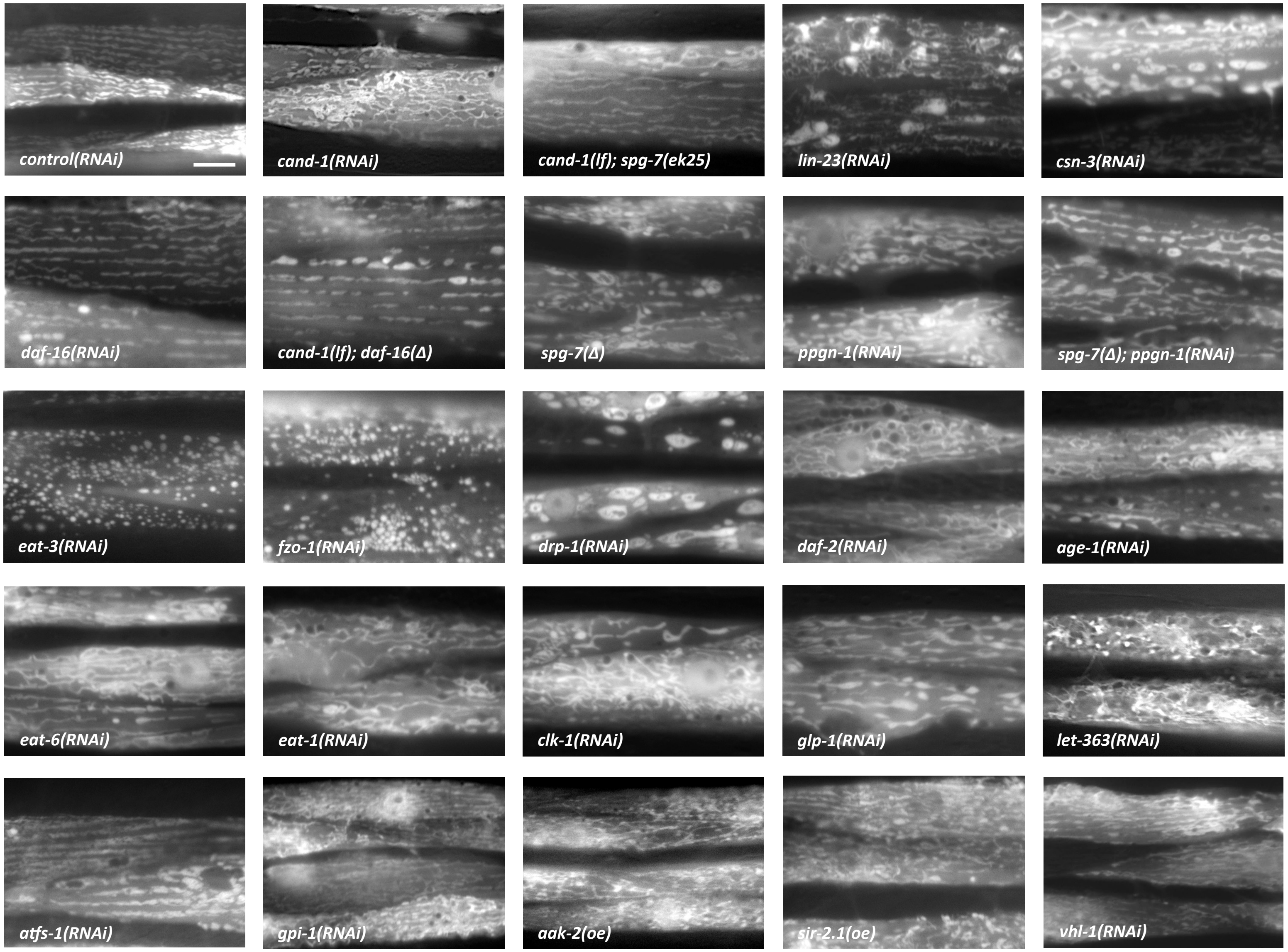

Supplementary Figure 5. Images of mitochondrial morphologies.
Representative images of mitochondria-targeted GFP in body wall muscles of the indicated mutants, overexpression strains, or RNAi-treated animals. Scale bar, 10 µm.

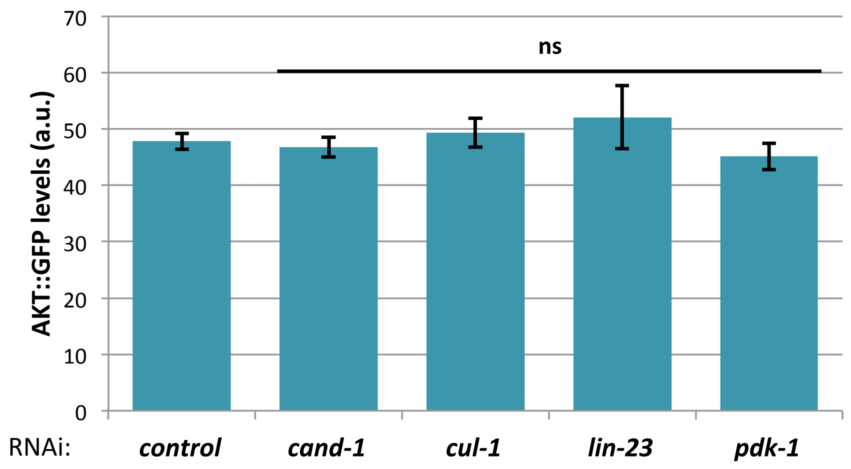

Supplementary Figure 6. AKT levels for mitochondrial fusion pathway inactivations.
The level of *Pakt-1*::AKT-1::GFP epifluorescence signal in the whole bodies of 20 L4/young-adult stage animals treated with the indicated RNAi. Error bars denote s.e.m. *P* values were determined by Student's t-test. *P* values for comparisons to control: ns = not significant.

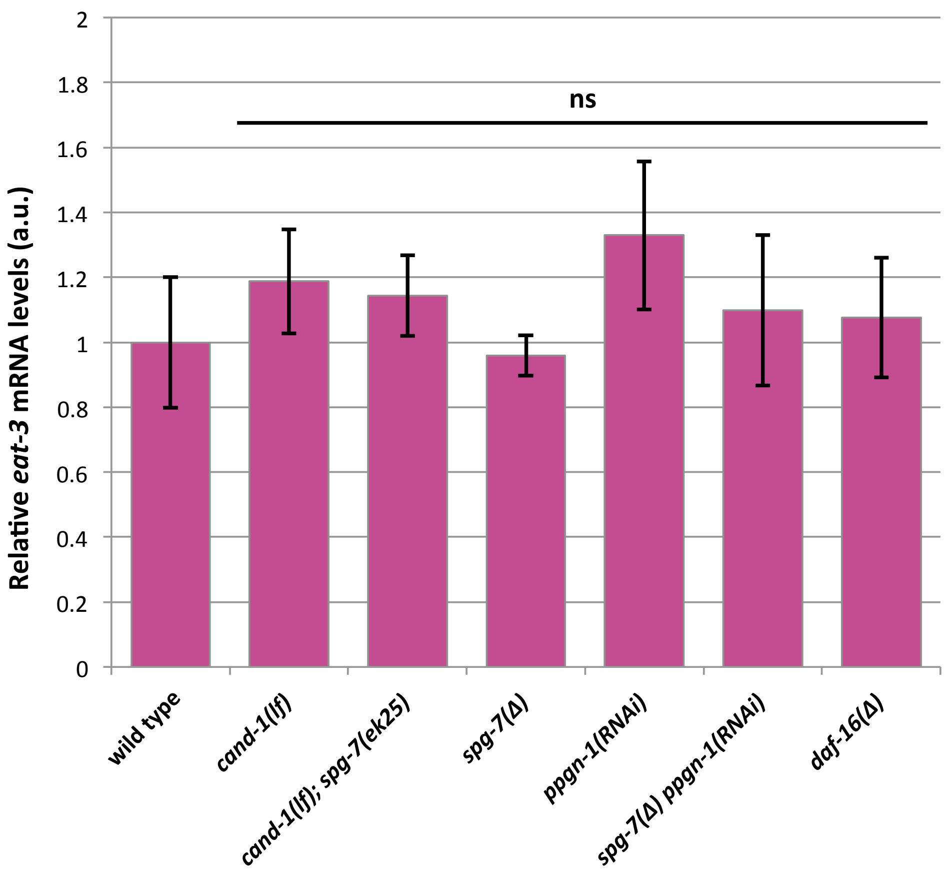

Supplementary Figure 7. ***eat-3*** **mRNA levels with mitochondrial fusion pathway inactivations.** RT-qPCR quantification of *eat-3* mRNA levels normalized to *rpl-19* control mRNA for young adults of the indicated genotypes/RNAi treatments. Error bars denote s.e.m. from two biological replicates, each with at least two technical replicates. *P* values were determined by Student's t-test. Asterisks above bars denote *P* values for comparisons to wild type; asterisks above lines denote comparisons under the lines: ns = not significant.

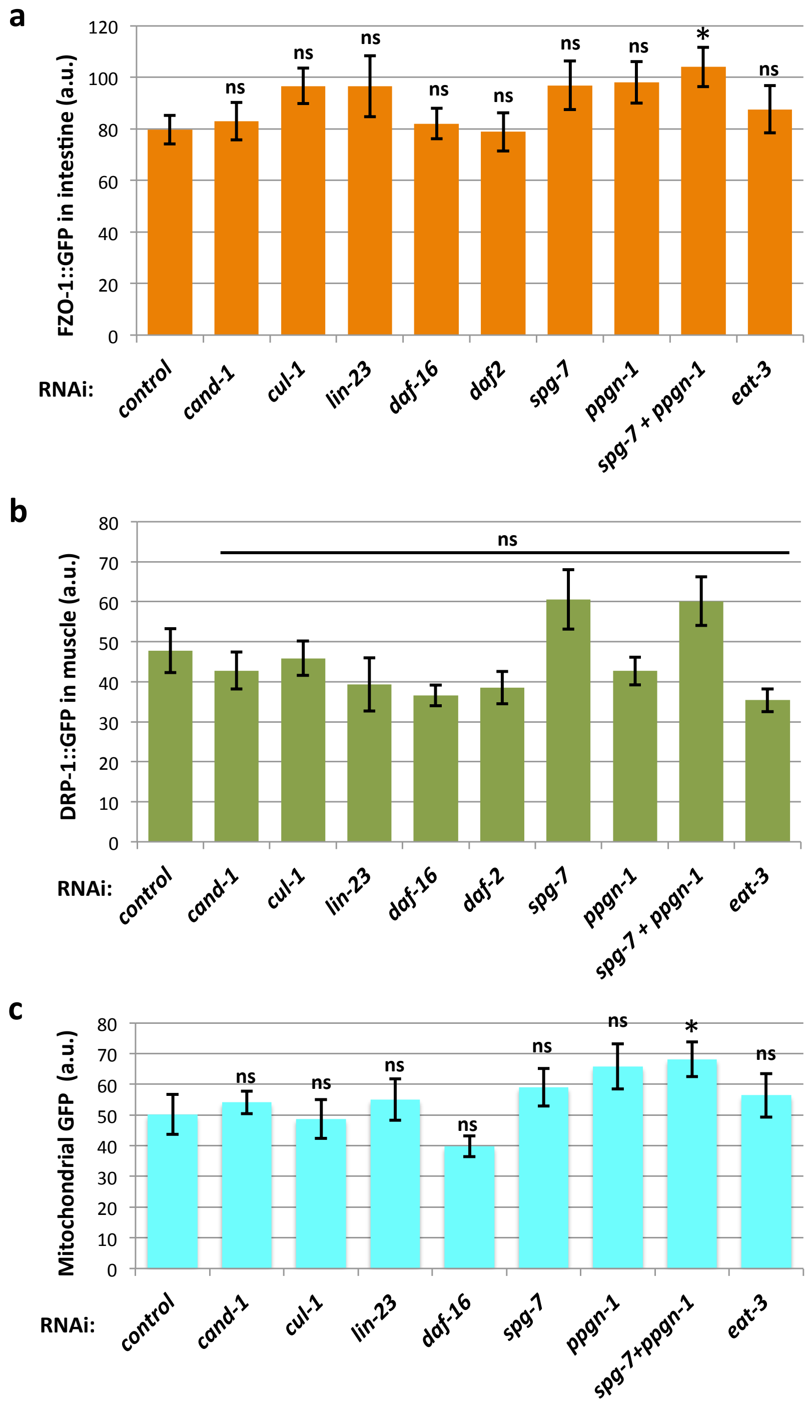

Supplementary Figure 8. DRP-1, FZO-1, and *Pmyo-3*::GFP(mit) levels for mitochondrial fusion pathway inactivations.

(**a**) The level of *Pmyo-3*::DRP-1::GFP signal was quantified in the body wall muscle cells of 20 L4/young-adult stage animals with the indicated RNAi treatments quantified from epifluorescence images. (**b**) The level of *Phsp*::FZO-1::GFP signal was quantified in intestine cells of 20 L4/young-adult stage animals with the indicated RNAi treatments quantified from epifluorescence images. (**c**) The level of *Pmyo-3*::GFP(mit) signal was quantified in the body wall muscle cells of 20 L4/young-adult stage animals with the indicated RNAi treatments quantified from epifluorescence images. Error bars denote s.e.m. from two biological replicates, each with at least two technical replicates. *P* values were determined by Student's t-test. *P* values for comparisons to controls: *P<0.05; ns = not significant.

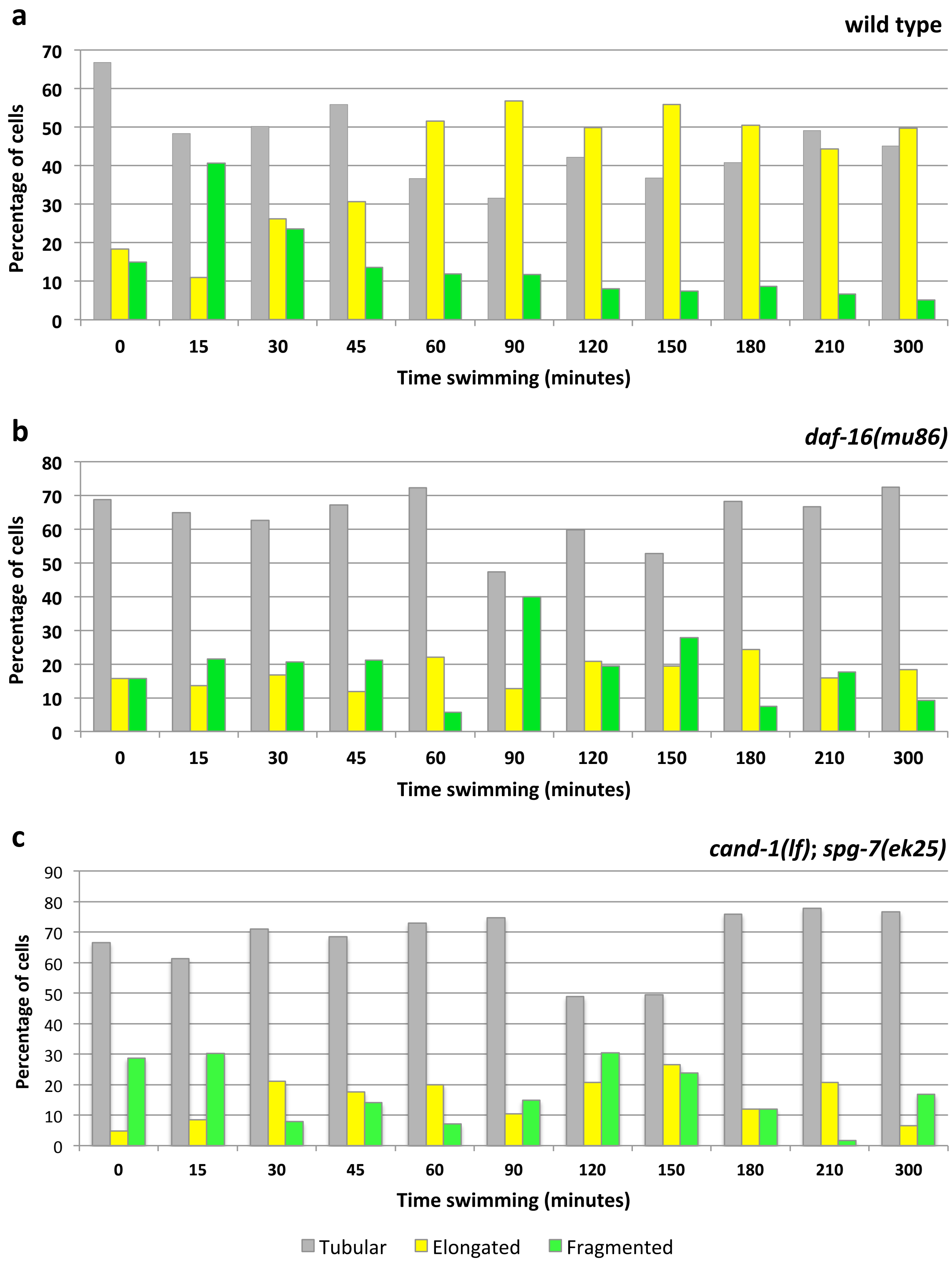

Supplementary Figure 9. Mitochondrial morphology in muscles during swimming. (**a**-**c**) The percentages of muscle cells with predominantly tubular, elongated, or fragmented mitochondria in wild type (a), *daf-16(mu86)* (b), and *cand-1*; *spg-7(ek25)* (c) animals upon induction of swimming behavior. All animals were fed OP50 bacteria for this experiment. Sample size (n) of muscle cells from left to right are: (**a**) wild type – 87; 118; 259; 111; 202; 111; 202; 111; 209; 95; 103; 253; 193. (**b**), *daf-16(mu86)* – 147; 279; 286; 261; 263; 313; 298; 284; 267; 296; 315. (**c**), *cand-1*; *spg-7(ek25)* – 230; 142; 114; 114; 155; 154; 125; 113; 83; 63; 77. Mitochondrial morphology was scored blinded.

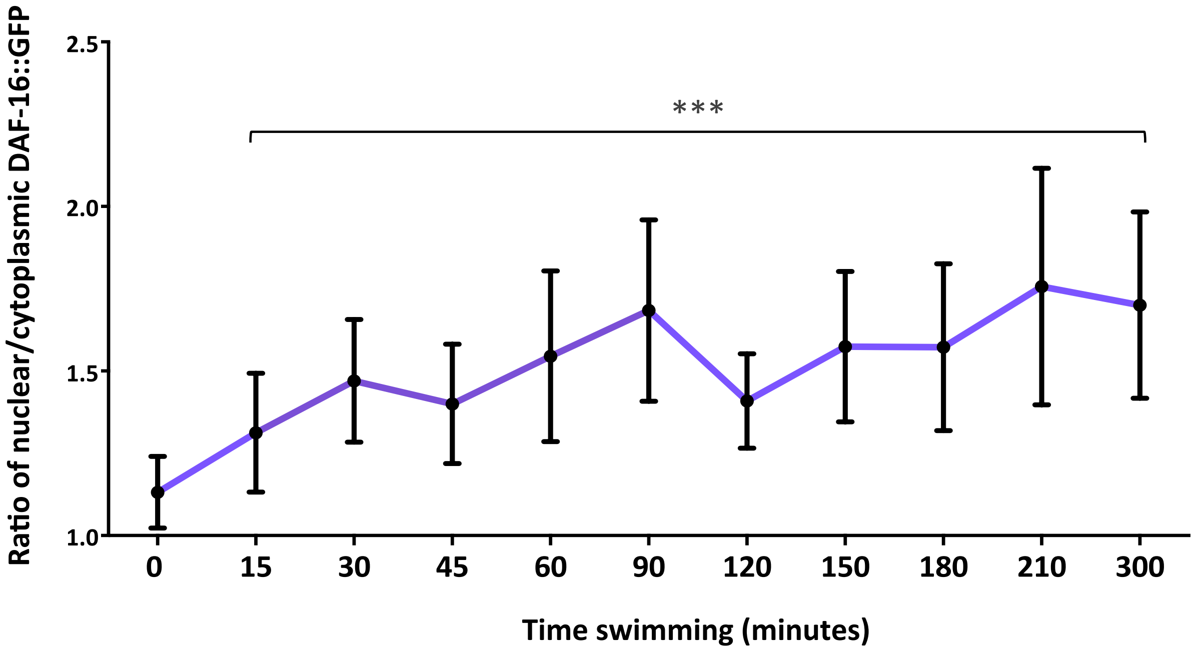

Supplementary Figure 10. DAF-16::GFP becomes nuclear localized during swimming. Quantification of the ratio of nuclear to cytoplasmic DAF-16::GFP intensity in 100 body wall muscle cells per time point after induction of swimming behavior. Error bars denote standard deviation. *P* values were determined by Student's t-test. *P* values for comparisons to the 0 time point: ****P*<0.001.

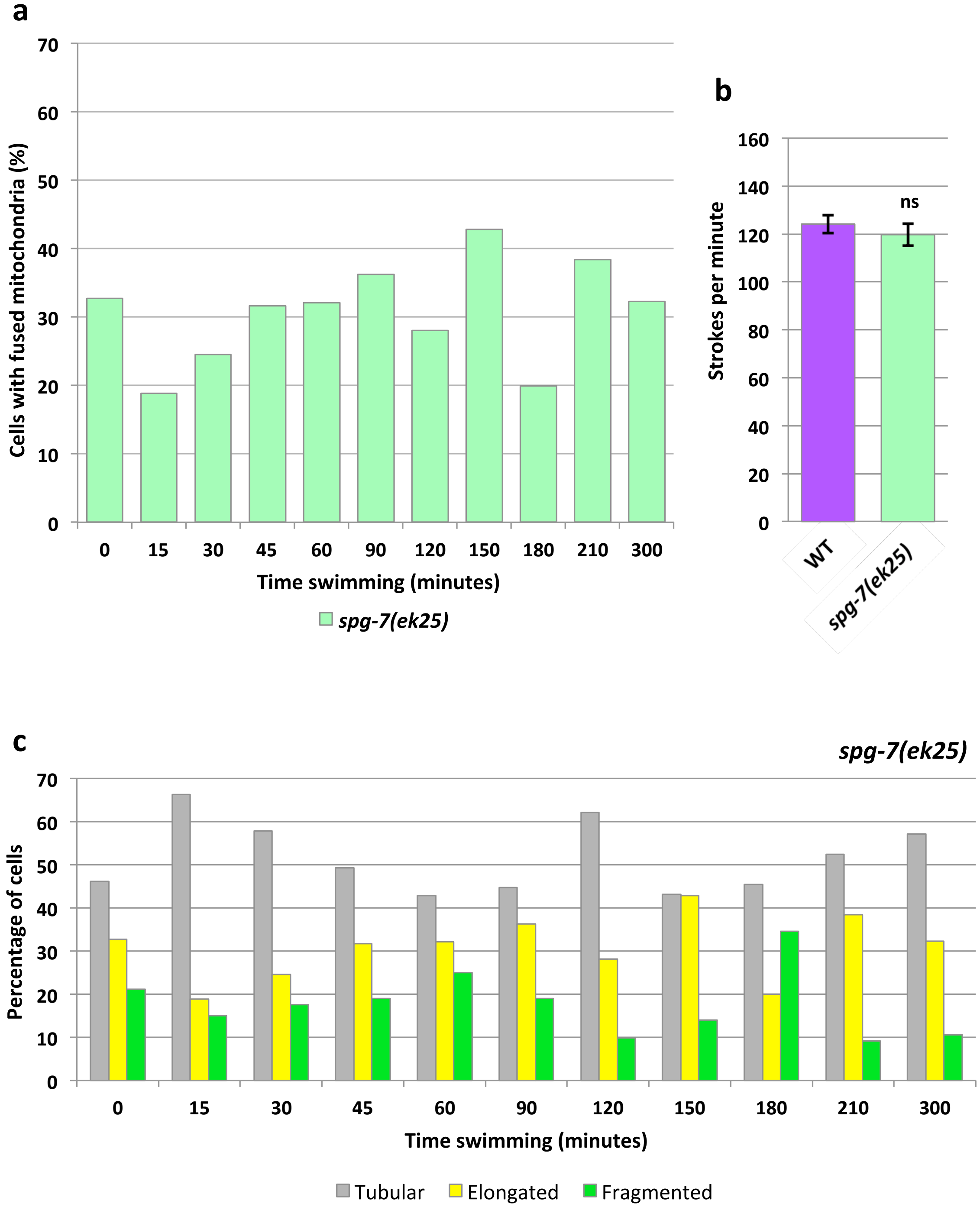

Supplementary Figure 11. Exercise does not induce mitochondrial fusion in *spg-7(ek25)*.
(**a**) The percentages of muscle cells with elongated mitochondria in *spg-7(ek25)* mutants for the indicated times post-induction of swimming behavior. (**b**) Average swim strokes-per-minute of 12 animals each for the indicated genotypes upon induction of swimming behavior. Error bars denote s.e.m. *P* values determined by Student's t-test: ns = not significant. The wild-type strokes-per-minute is from Fig. 5b, and is shown here for comparison. (**c**) The percentages of muscle cells with predominantly tubular, elongated, or fragmented mitochondria in *spg-7(ek25)* animals upon induction of swimming behavior. Sample size (n) of muscle cells from left to right are: 260; 287; 258; 300; 296; 425; 253; 264; 341; 263; 313. Mitochondria were scored blinded.

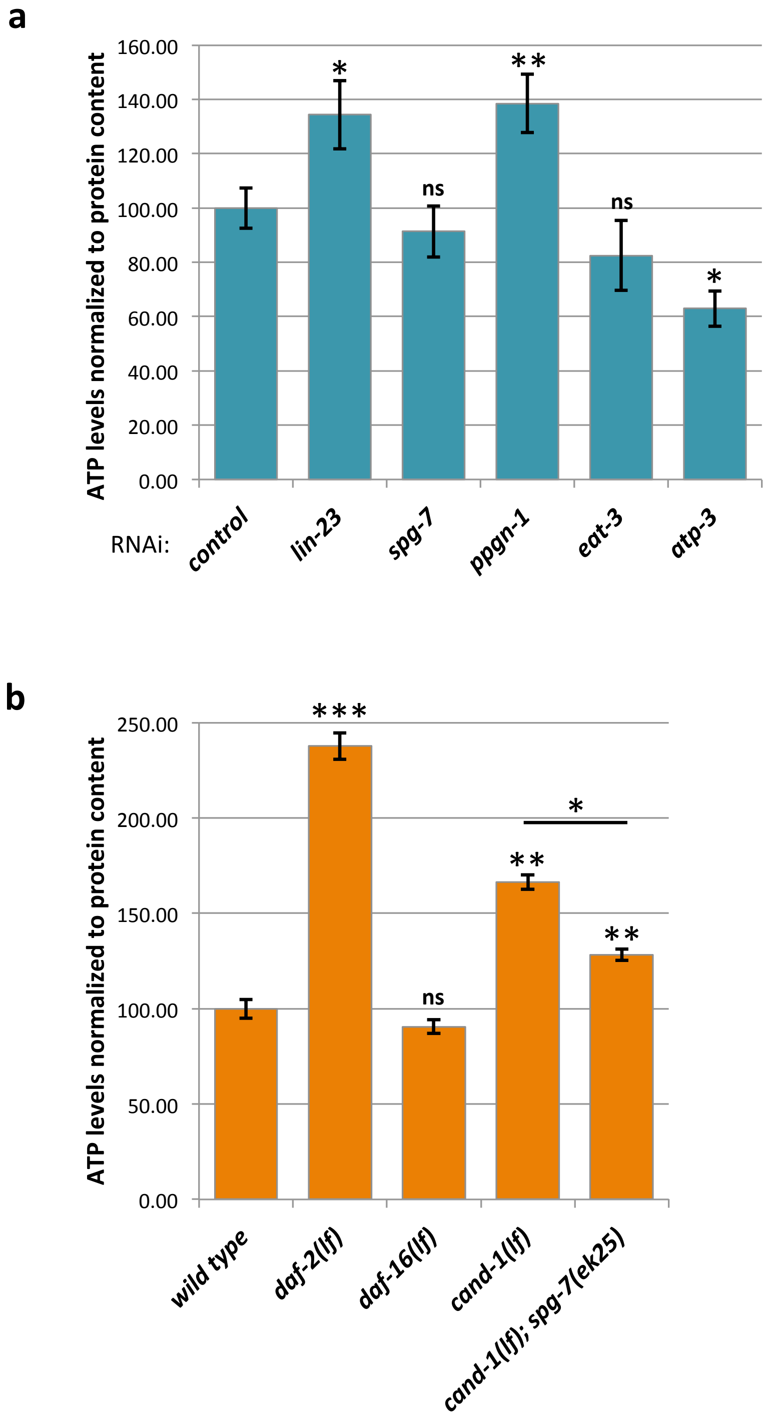

Supplementary Figure 12. ATP levels are elevated in animals with elongated mitochondria. The ratio of ATP to whole-animal protein level is shown for the indicated RNAi treatments (**a**) or mutant genotypes (**b**) for L4/young-adult stage animals. Levels are standardized to control RNAi or wild type (set to 100). *atp-3* RNAi is a negative control that depletes a component of the ATP synthase complex V. Error bars denote s.e.m. from two biological replicates, each with three technical replicates. *P* values were determined by Student's t-test. Asterisks above bars denote *P* values for comparisons to wild type/controls; asterisks above lines denote comparisons under the lines: *$P<0.05$; **$P<0.01$; ***$P<0.001$; ns = not significant.

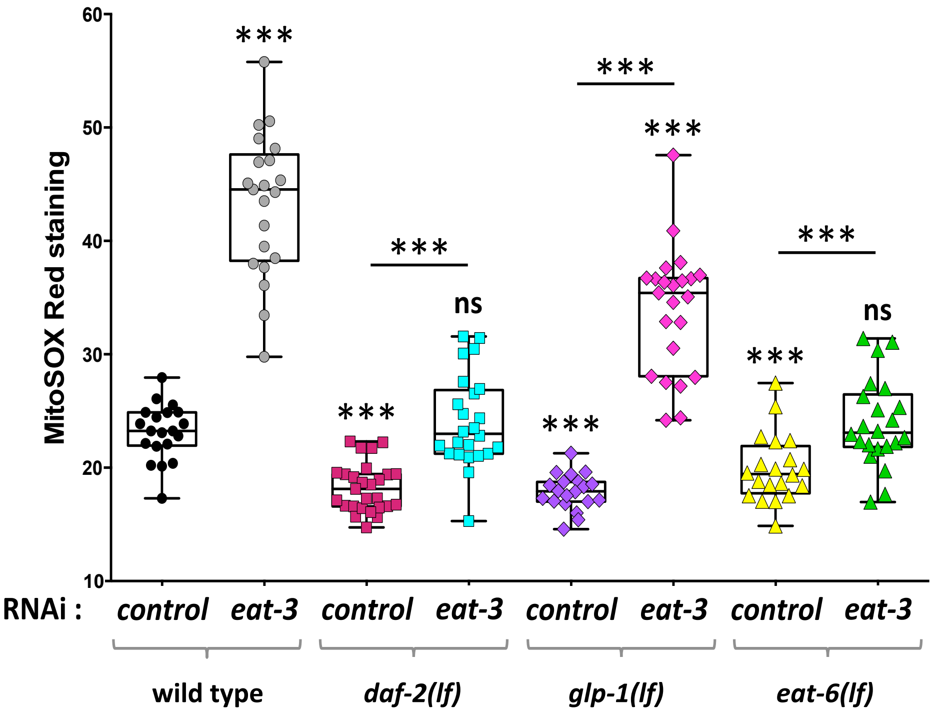

Supplementary Figure 13. Mitochondrial ROS in longevity mutants upon *eat-3* RNAi. Mean intensity of the posterior pharyngeal bulb stained with the ROS-indicator MitoSOX Red in 20 young adults of wild type, *daf-2(e1370)*, *glp-1(e2141)*, and *eat-6(ad467)* genotypes subjected to control or *eat-3* RNAi. See Supplementary Table 4 for statistics. Boxes represent the s.e.m. range with the mean denoted by a central horizontal line; vertical lines extending above and below the box denote the range. Asterisks above bars denote *P* values for comparisons to wild type/controls; asterisks above lines denote comparisons under the lines: ***P<0.001; ns = not significant.

Supplementary Figure 14. Uncropped western blot scans.
Uncropped blots are shown for Figure 2c (**a**); 2d (**b**); and 2f (**c**).

a

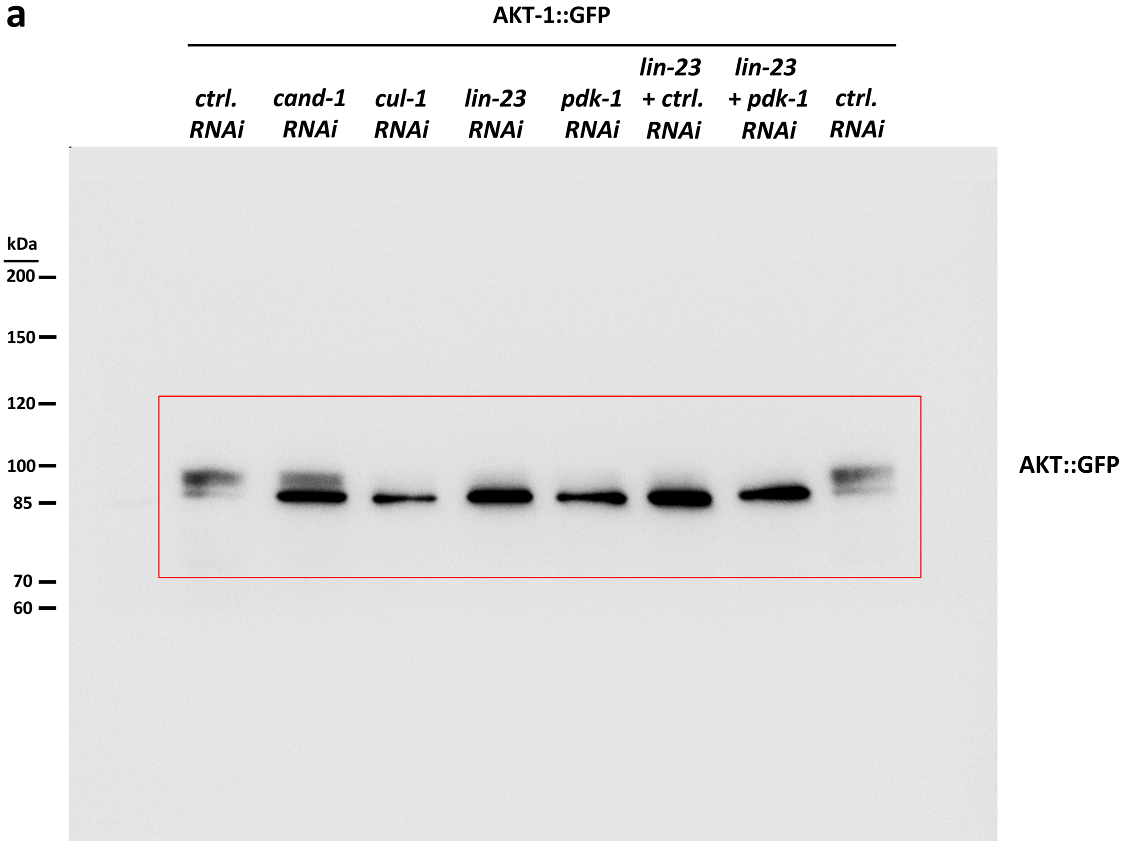

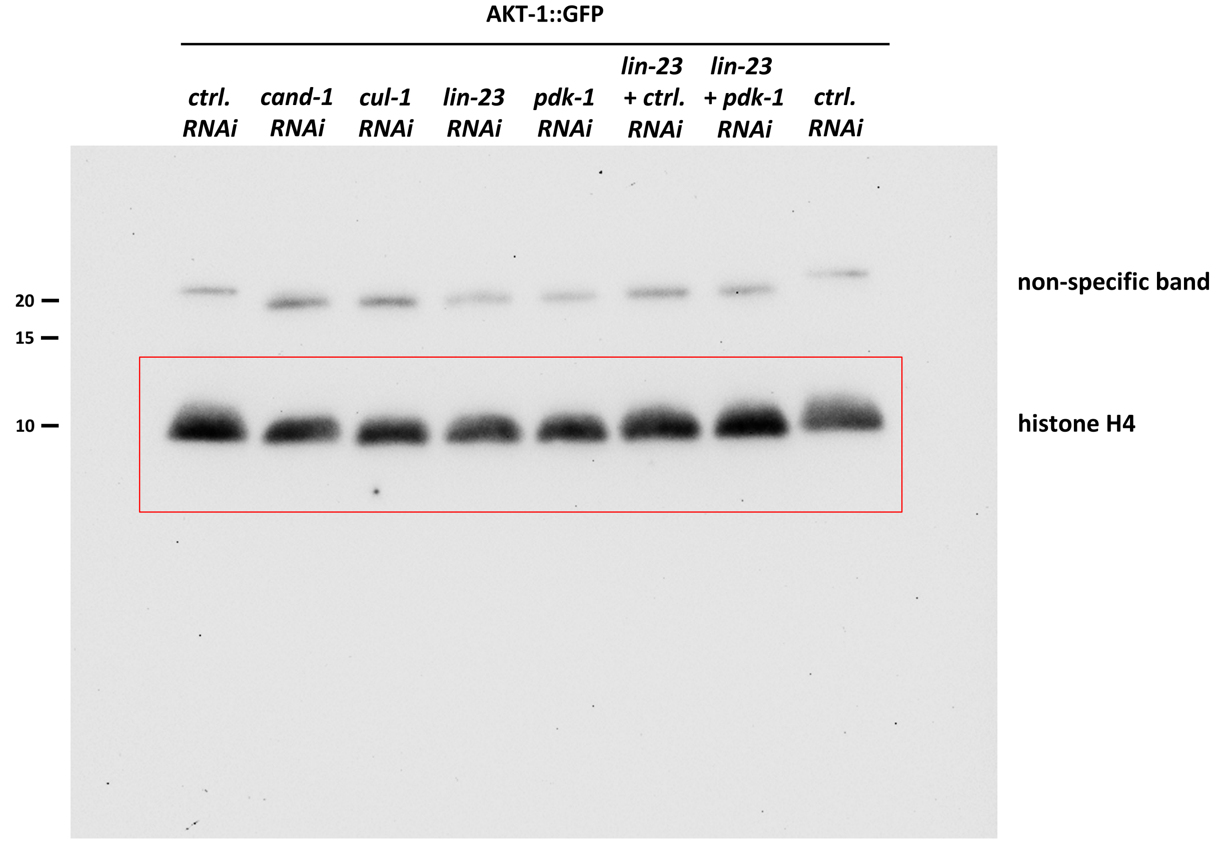

b

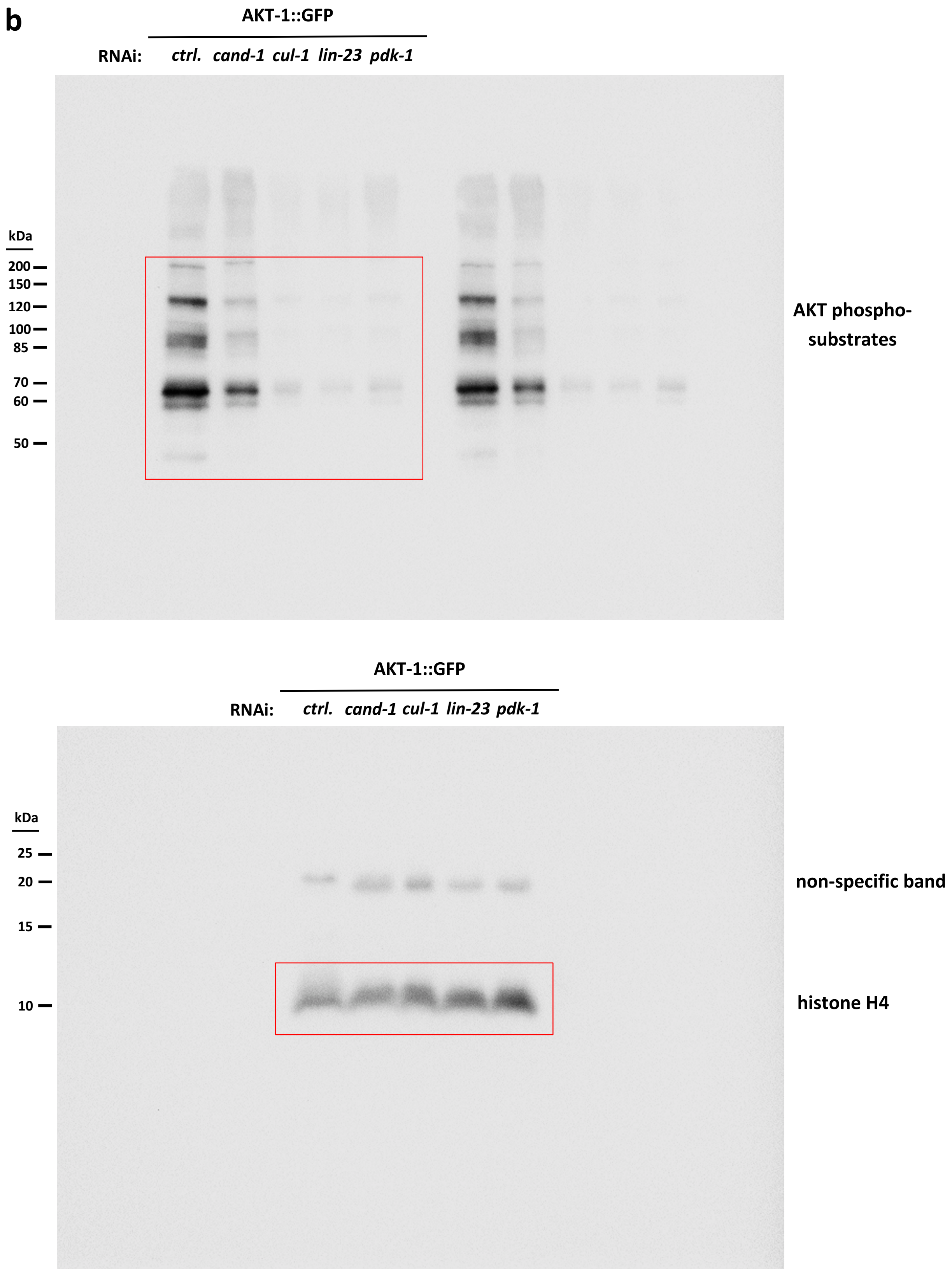
AKT-1::GFP
RNAi: ctrl. cand-1 cul-1 lin-23 pdk-1
kDa
200
150
120
100
85
70
60
50
AKT phospho-substrates
AKT-1::GFP
RNAi: ctrl. cand-1 cul-1 lin-23 pdk-1
kDa
25
20
15
10
non-specific band
histone H4

c

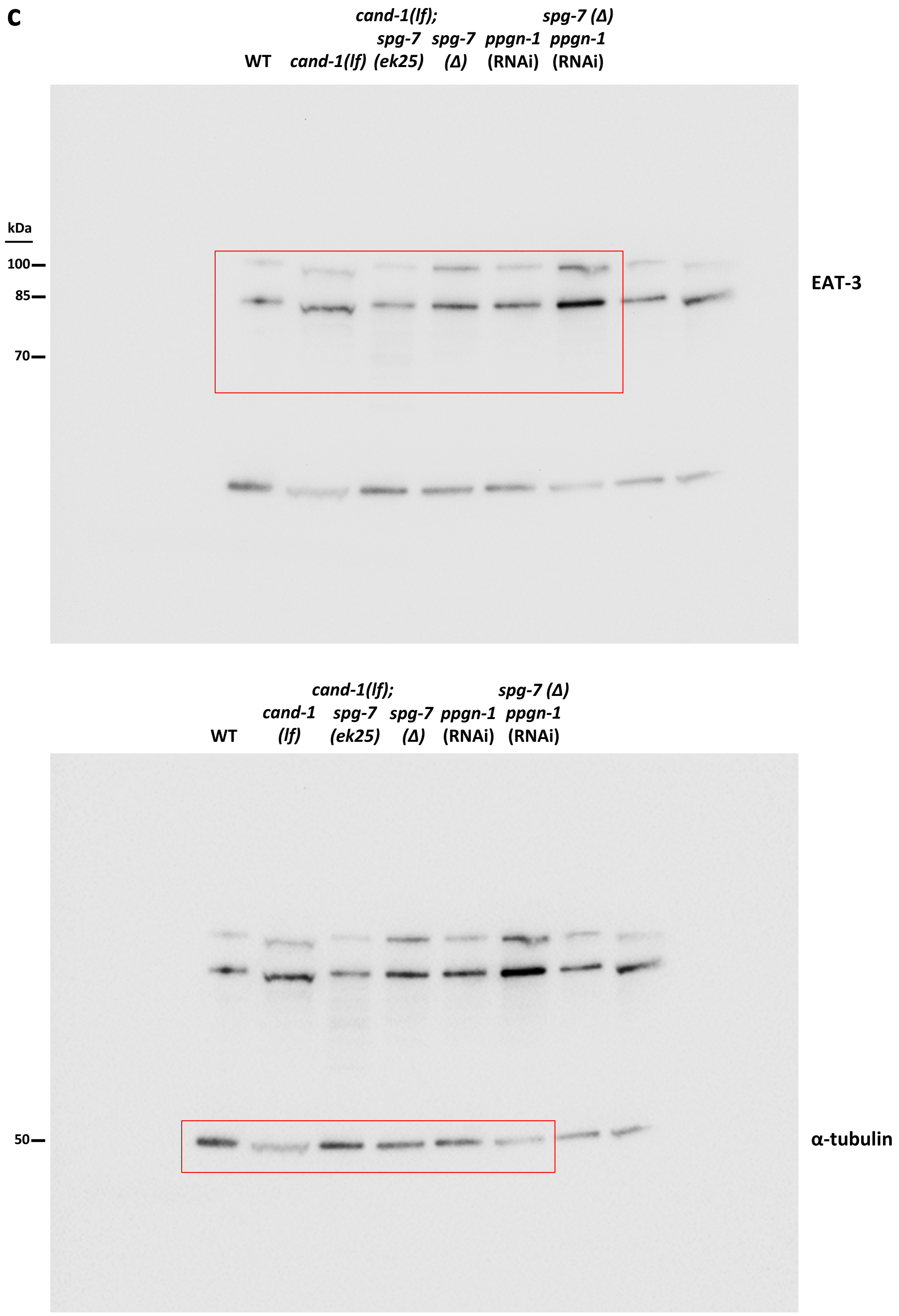
WT
cand-1(lf)
cand-1(lf); spg-7 (ek25)
spg-7 (Δ)
ppgn-1 (RNAi)
spg-7 (Δ) ppgn-1 (RNAi)
kDa
100
85
70
EAT-3
WT
cand-1 (lf)
cand-1(lf); spg-7 (ek25)
spg-7 (Δ)
ppgn-1 (RNAi)
spg-7 (Δ) ppgn-1 (RNAi)
50
α-tubulin

Supplementary Table 1. Analysis of RNAi of *ek25*-candidates genes for *cand-1* mutant phenotypes

RNAi	bobtail phenotype (%)			egg hatch (%)		
	cand-1	***cand-1*; *ek25***	**wild type**	***cand-1***	***cand-1*; *ek25***	**wild type**
control[b]	11.0	1.2	0	94.6	100.0	100.0
cand-1[a]	29.0	2.0	0	58.0	91.0	97.0
csn-3[a,c]	dead eggs	4.0	0	0.0	96.0	56.0
spg-7[a]	40.7	11.4	0	sterile	sterile	sterile
spg-7:ctrl 1:1[a]	48.0	6.0	0	sterile	45.4	sterile
spg-7:ctrl 1:2[a]	23.0	4.0	0	sterile	77.7	81.8
spg-7:ctrl 1:5[a]	11.0	5.0	0	75.6	95.2	91.0
Y47G6A.15[a]	12.0	1.0	0	92.7	98.0	100.0

[a]n = ~100 animals;
[b]n = ~250 animals;
[c]*csn-3* RNAi acts as an enhancer for *cand-1* mutants that results in 100% dead eggs.

Supplementary Table 2. Lifespan analysis

Genotype/(RNAi Treatment)	n (censored)	median life span	mean life span ± STDEV	*P* value vs. wild type		*P* value vs. control	
				Log-rank	Wilcoxon	Log-rank	Wilcoxon
N2 wild type (*control* RNAi)	86 (5)	14±0.2	15.5±0.2	–	–	–	–
N2 (*eat-3* RNAi)	89 (1)	15±0.1	15.4	0.8660	0.9684	–	–
N2 (*cand-1* RNAi)	75 (10)	22±0.3	25.4	< 0.0001	< 0.0001	–	–
N2 (*lin-23* RNAi)	129 (14)	18±0.3	19.2	0.0002	0.0090	–	–
N2 (*spg-7* RNAi)	103 (5)	13±0.2	15.3	0.3153	0.4344	–	–
N2 (*ppgn-1* RNAi)	108 (2)	19±0.1	20.2	< 0.0001	< 0.0001	–	–
cand-1(tm1683) (*control* RNAi)	76 (9)	17±0.3	19.7	0.0015	0.0109	–	–
cand-1(tm1683); *spg-7(ek25)* (*control* RNAi)	52 (1)	14±0.1	16.0	0.3879	0.8882	–	–
N2 wild type (*control* RNAi)	56 (8)	13±0.3	15.0	–	–	–	–
spg-7(tm2312) (*control* RNAi)	81(2)	20±0.2	17.7	0.0003	0.0076		
N2 (*spg-7* + *ppgn-1* RNAi)	118(1)	19±0.1	20.4	< 0.0001	< 0.0001		
N2 wild type (*control* RNAi)	101 (8)	17±0.3	18.0	–	–	–	–
N2 (*eat-3* RNAi)	97 (0)	15±0.1	15.2	0.8184	0.6745	–	–
daf-2(e1370) (*control* RNAi)	109 (8)	41±0.1	37.8	< 0.0001	< 0.0001	< 0.0001	< 0.0001

daf-2(e1370) (*eat-3* RNAi)	87 (0)	15±0.0	18.3	0.1265	0.3773		
eat-2(ad116) (*control* RNAi)	43 (0)	23±0.0	22.3	< 0.0001	< 0.0001	< 0.0001	< 0.0001
eat-2(ad116) (*eat-3* RNAi)	67 (0)	11±0.0	11.6	< 0.0001	< 0.0001		
eat-6(ad467) (*control* RNAi)	80 (0)	24±0.2	24.6	< 0.0001	< 0.0001	< 0.0001	< 0.0001
eat-6(ad467) (*eat-3* RNAi)	57 (0)	13±0.0	13.4	0.0112	0.0490		
glp-1(e2141) (*control* RNAi)	65 (4)	22±0.2	23.4	< 0.0001	< 0.0001	< 0.0001	< 0.0001
glp-1(e2141) (*eat-3* RNAi)	80 (2)	13±0.2	15.3	0.7682	0.6498		
N2 (*clk-1* RNAi)	66 (2)	17±0.1	18.5	< 0.0001	< 0.0001	–	–
N2 (*clk-1* + *control* RNAi)	86 (5)	21±0.2	22.2	< 0.0001	< 0.0001	< 0.0001	< 0.0001
N2 (*clk-1* + *eat-3* RNAi)	157 (5)	15±0.1	16.5	0.0008	0.0017		
sir-2.1(oe) (*control* RNAi)	122 (4)	21±0.1	21.9	< 0.0001	< 0.0001	< 0.0001	< 0.0001
sir-2.1(oe) (*eat-3* RNAi)	126 (1)	16±0.1	15.2	0.0815	0.0681		
N2 (*let-363* RNAi)	52 (10)	19±0.3	20.6	< 0.0001	< 0.0001	–	–
N2 (*let-363* + *control* RNAi)	40 (24)	17±0.4	21.5	0.0081	0.0109	0.0322	0.1002
N2 (*let-363* + *eat-3* RNAi)	48 (4)	15±0.2	15.0	0.5964	0.4065		
aak-2(oe) (*control* RNAi)	33 (15)	21±0.4	25.0	< 0.0001	< 0.0001	0.0081	0.0091
aak-2(oe) (*eat-3* RNAi)	42 (1)	15±0.1	16.0	0.0367	0.1029		
N2 (*gpi-1* RNAi)	67 (14)	17±0.3	18.0	0.0004	0.0003	–	–

N2 (*gpi-1* + *control* RNAi)	99 (7)	17±0.2	18.5	< 0.0001	0.0008	< 0.0001	0.0082
N2 (*gpi-1* + *eat-3* RNAi)	98 (5)	14±0.2	15.0	0.9975	0.1816		
N2 wild type (*control* RNAi)	109 (22)	14±0.3	13.6	–	–	–	–
vhl-1(ok161) (*control* RNAi)	112 (2)	22±0.1	23.0	< 0.0001	< 0.0001	0.1691	0.4245
vhl-1(ok161) (*eat-3* RNAi)	99 (0)	22±0.0	21.2	< 0.0001	< 0.0001		
N2 (*fzo-1* RNAi)	126 (2)	10±0.1	10.6	< 0.0001	< 0.0001	–	–
daf-2(e1370) (*control* RNAi)	94 (13)	39±0.3	37.7	< 0.0001	< 0.0001	< 0.0001	< 0.0001
daf-2(e1370) (*fzo-1* RNAi)	121(0)	21±0.0	22.0	< 0.0001	< 0.0001		
N2 wild type (*control* RNAi)	71 (2)	14±0.1	13.8	–	–	–	–
atfs-1(et17) (*control* RNAi)	87 (13)	11±0.3	12.3	0.0015	0.0085	–	–
atfs-1(et17) (*cand-1* RNAi)	67 (2)	13±0.1	13.6	0.4160	0.7258	0.0083	0.0036
atfs-1(et17) (*lin-23* RNAi)	97 (15)	15±0.3	16.0	0.1044	0.1027	< 0.0001	< 0.0001
atfs-1(et17) (*control* RNAi)	99 (2)	9±0.1	9.0	< 0.0001	< 0.0001	–	–
atfs-1(et17) (*spg-7* RNAi)	107 (14)	19±0.3	18.5	< 0.0001	< 0.0001	–	–
atfs-1(et17) (*ppgn-1* RNAi)	56 (23)	17±0.4	18.3	< 0.0001	< 0.0001	–	–
atfs-1(et17) (*spg-7* + *ppgn-1* RNAi)	115 (7)	19±0.2	19.8	< 0.0001	< 0.0001	–	–

n = number of animals scored for lifespan experiment (censored animals were included in the Log-rank and Wilcoxon statistical analysis).

Supplementary Table 3. Lifespan analysis-Swim assay

Genotype/(RNAi Treatment)	n (censored)	median life span ± STDEV	mean life span ± STDEV	*P* value vs. wild type		*P* value vs. control	
				Log-rank	Wilcoxon	Log-rank	Wilcoxon
N2 wild type (crawl)	91 (11)	10±0.3	12.4	–	–	–	–
N2 wild type (swim regimen A)	99 (29)	16±0.4	18.8	–	–	< 0.0001[a]	< 0.0001[b]
N2 wild type (crawl)	114 (7)	13±0.2	13.0	–	–	–	–
N2 wild type (swim regimen B)	145 (12)	15±0.2	17.4	–	–	< 0.0001[c]	< 0.0001[d]
N2 wild type (swim regimen C)	118 (32)	19±0.4	20.6	–	–	< 0.0001[c]	< 0.0001[c]
N2 wild type (crawl)	200 (16)	12±0.2	12.0	–	–	–	–
N2 wild type (swim regimen C)	197 (46)	15±0.3	16.3	–	–	< 0.0001[c]	< 0.0001[c]
cand-1(tm1683); *spg-7(ek25)* (crawl)	100 (24)	10±0.3	10.2	0.0370	< 0.0001	–	–
cand-1(tm1683); *spg-7(ek25)* (swim regimen C)	80 (26)	8±0.4	10.0	0.0008	< 0.0001	0.5631	0.6764
daf-16(mu86) (crawl)	131 (8)	10±0.2	11.9	0.2448	0.1217	–	–
daf-16(mu86) (swim regimen C)	80 (3)	12±0.1	13.2	< 0.0001	0.0368	0.1228	0.0307

n = number of animals scored for lifespan experiment (censored animals were included in the Log-rank and Wilcoxon statistical analysis)

[a,b]Compared to N2 wild-type lifespan (combined from all non-swim experiments, the *P* values are 0.6288 and 0.1138 for Log-Rank and Wilcoxon tests, respectively.

[c]Compared to N2 wild-type lifespan (combined from all non-swim experiments), the *P* values are <0.0001 for Log-Rank and Wilcoxon tests.

[d]Compared to N2 wild-type lifespan (combined from all non-swim experiments), the *P* value is 0.0041 for Wilcoxon test.

Supplementary Table 4. MitoSOX Red staining analysis

<table>
<tr><th>Genotype/(RNAi Treatment)</th><th>n</th><th>mean
± s.e.m.</th><th>P value
vs. wild type</th><th>P value
vs. control RNAi</th></tr>
<tr><td>N2 wild type (control RNAi)</td><td>20</td><td>23.15±0.5</td><td>–</td><td>–</td></tr>
<tr><td>N2 (eat-3 RNAi)</td><td>21</td><td>43.32±1.3</td><td>< 0.0001</td><td>–</td></tr>
<tr><td>daf-2(e1370) (control RNAi)</td><td>27</td><td>18.25±0.4</td><td>< 0.0001</td><td rowspan="2">< 0.0001</td></tr>
<tr><td>daf-2(e1370) (eat-3 RNAi)</td><td>24</td><td>24.06±0.8</td><td>0.3855</td></tr>
<tr><td>glp-1(e2141) (control RNAi)</td><td>20</td><td>17.85±0.3</td><td>< 0.0001</td><td rowspan="2">< 0.0001</td></tr>
<tr><td>glp-1(e2141) (eat-3 RNAi)</td><td>23</td><td>33.94±1.1</td><td>< 0.0001</td></tr>
<tr><td>eat-6(ad467) (control RNAi)</td><td>20</td><td>19.91±0.6</td><td>0.0006</td><td rowspan="2">0.0006</td></tr>
<tr><td>eat-6(ad467) (eat-3 RNAi)</td><td>22</td><td>23.92±0.8</td><td>0.4513</td></tr>
</table>

n = number of animals imaged for MitoSOX Red staining.

Supplementary References

1. Furuyama, T., Nakazawa, T., Nakano, I. & Mori, N. Identification of the differential distribution patterns of mRNAs and consensus binding sequences for mouse DAF-16 homologues. *The Biochemical journal* **349**, 629-634 (2000).
2. Tepper, R. G., Ashraf, J., Kaletsky, R., Kleemann, G., Murphy, C. T. & Bussemaker, H. J. PQM-1 complements DAF-16 as a key transcriptional regulator of DAF-2-mediated development and longevity. *Cell* **154**, 676-690 (2013).
3. Gerstein, M. B. *et al.* Integrative analysis of the Caenorhabditis elegans genome by the modENCODE project. *Science* **330**, 1775-1787 (2010).
